# Machine Learning Triage of LDLR Variants of Uncertain Significance Using Predictor Concordance and ACMG-Aligned Evidence Mapping

**DOI:** 10.3390/genes17091057

**Published:** 2026-08-31

**Authors:** BalaSubramani Gattu Linga, Faisal E. Ibrahim, Nader Al-Dewik

**Affiliations:** 1 Department of Research, Women’s Wellness and Research Center, Hamad Medical Corporation (HMC), Doha 3050, Qatar; blinga@hamad.qa (B.G.L.); fibrahim23@hamad.qa (F.E.I.); 2 Translational and Precision Medicine Research Facility, Women’s Wellness and Research Center, Hamad Medical Corporation (HMC), Doha 3050, Qatar; 3 Translational Research Institute (iTRI), Hamad Medical Corporation (HMC), Doha 3050, Qatar; 4 Qatar Preterm and Precision Medicine Research Clinic, Women’s Wellness and Research Center, Hamad Medical Corporation (HMC), Doha 3050, Qatar; 5 Genomics and Precision Medicine (GPM), College of Health & Life Science (CHLS), Hamad Bin Khalifa University (HBKU), Doha 34110, Qatar; 6 Faculty of Health and Social Care Sciences, Kingston University, St. George’s University of London, London KT1 2EE, UK

**Keywords:** LDLR, familial hypercholesterolemia, variants of uncertain significance, ClinVar, machine learning, ACMG/AMP

## Abstract

**Background:** Variants of uncertain significance (VUS) in the *LDLR* gene remain a major barrier to the molecular diagnosis of familial hypercholesterolemia. Although computational approaches offer scalable prioritization, their clinical utility is limited by predictor discordance, incomplete annotations, and inflated performance arising from variant-type imbalance. We aimed to develop a calibrated, uncertainty-aware machine learning framework for *LDLR* VUS triage. **Methods:** We developed an end-to-end pipeline integrating multi-source variant annotations and 45 engineered features (expanded to 68 encoded features) to train RandomForest, XGBoost, and support vector machine models. The framework incorporates predictor concordance analysis, splice-aware annotation, and explicit uncertainty modeling. Models were trained using nested cross-validation and evaluated on an independent ClinGen FH-VCEP expert-panel dataset. **Results:** The XGBoost model achieved high discriminative performance (ROC-AUC up to 0.99 for all variants and ~0.97 for missense variants). Among 1092 *LDLR* VUS, 69% were assigned a directional classification, including 485 (44.4%) pathogenic-leaning and 268 (24.5%) benign-leaning variants. The remaining 31% were explicitly categorized as predictor-discordant (n = 170, 15.6%) or unresolved (n = 169, 15.5%), preserving uncertainty. Predictor discordance was particularly enriched among missense variants, indicating that naive aggregation of in silico predictors may lead to overclassification. **Conclusions:** This framework provides a scalable, transparent, and clinically aligned approach for *LDLR* VUS prioritization, generating ACMG/AMP-compatible computational evidence to support expert curation and downstream functional validation.

## 1. Introduction

Familial hypercholesterolemia (FH; OMIM #143890) is one of the most common inherited metabolic disorders, with an estimated heterozygote prevalence of approximately 1 in 300 individuals worldwide [1]. Despite this relatively high prevalence, FH remains substantially underdiagnosed and undertreated, with a large proportion of affected individuals lacking a definitive molecular diagnosis. This diagnostic gap delays the initiation of lipid-lowering therapy and increases the risk of premature atherosclerotic cardiovascular disease. Pathogenic variants in the low-density lipoprotein receptor gene (*LDLR*; OMIM 606945; UniProt P01130) account for approximately 85–90% of genetically confirmed FH cases [2,3,4,5], making accurate interpretation of *LDLR* variants central to diagnosis, risk stratification, and cascade screening of relatives [4,6].

Variant interpretation in *LDLR* is complicated by the gene’s structural and functional complexity, which includes multiple ligand-binding repeats, epidermal growth factor–like domains, and regulatory regions that govern receptor trafficking and recycling. Variants across these domains can exert heterogeneous effects on receptor expression, LDL binding, and cellular uptake, further complicating clinical classification. Public repositories such as ClinVar aggregate thousands of *LDLR* variants, many of which remain classified as variants of uncertain significance (VUS). In clinical practice, VUS represent a major bottleneck: they limit diagnostic certainty, complicate cascade screening strategies, and introduce ambiguity in patient management. Although the ClinGen Familial Hypercholesterolemia Variant Curation Expert Panel (FH-VCEP) has established *LDLR*-specific ACMG/AMP classification guidelines, applying these criteria requires integration of diverse evidence types—including functional assays, segregation data, and population frequencies, which are frequently incomplete or unavailable for rare variants [7,8,9].

Computational approaches offer a scalable solution for prioritizing VUS prior to formal ACMG/AMP evaluation [8]. Widely used in silico predictors, including REVEL, AlphaMissense, CADD, PolyPhen-2, and SIFT, demonstrate strong discriminative performance in benchmark settings, and recent efforts have calibrated REVEL to ACMG/AMP evidence strengths [10,11,12,13,14,15]. However, several limitations constrain their clinical utility. First, model performance can be inflated by consequence-driven signals, as pathogenicity labels in ClinVar are strongly correlated with variant class, leading to overestimation of performance in heterogeneous datasets. Second, naive aggregation of multiple predictors can produce overconfident classifications when tools disagree, obscuring biologically meaningful uncertainty. Third, incomplete annotation coverage may result in confident predictions based on limited or missing evidence, without explicit representation of uncertainty [16].

To address these challenges, we developed an uncertainty-aware machine learning framework for *LDLR* VUS prioritization. The proposed pipeline integrates multi-source annotations, consequence-stratified evaluation, explicit modeling of predictor concordance, and splice-aware annotation for non-coding variants. Importantly, the framework preserves uncertainty by distinguishing concordant, discordant, and limited-evidence cases, rather than forcing binary classification. Model outputs are further calibrated and mapped to ACMG/AMP-compatible PP3/BP4 evidence strengths, enabling direct clinical interpretability. We evaluated the framework using nested cross-validation and independent validation against ClinGen FH-VCEP expert panel-reviewed variants, providing a robust assessment of performance in a clinically relevant setting. 

## 2. Methods

### 2.1. Data Source and Label Normalization

This section describes the source of variant data and how clinical significance labels were standardized for model training. A total of 3962 *LDLR* variant records were retrieved from ClinVar [9]. Each record included genomic coordinates, molecular consequence (MC), variant class (CLNVC), HGVS nomenclature, clinical significance (CLNSIG), allele origin (ORIGIN), review status (CLNREVSTAT), and population allele frequency annotations derived from ESP, ExAC, and the 1000 Genomes Project. ClinVar significance annotations were harmonized into three standardized classes: P_LP (Pathogenic, Likely_pathogenic; n = 1880), B_LB (Benign, Likely_benign; n = 990), and VUS (Uncertain_significance and any unmapped or conflicting labels; n = 1092). This normalization ensured consistent downstream model training and evaluation while preserving clinically relevant distinctions between labeled and unlabeled variants. To support unbiased external validation, variants were additionally stratified based on review status (CLNREVSTAT), distinguishing expert panel-reviewed records from single- or multi-submitter entries. Variants curated by the ClinGen FH-VCEP expert panel were excluded from model training and reserved for independent validation, ensuring orthogonality between training and evaluation datasets.

### 2.2. In Silico Annotation

This section details the computational tools used to annotate each variant with predicted functional impact scores. Model development, calibration, and evaluation were implemented as modular and reproducible stages within a unified pipeline. For each variant, in silico predictor annotations were retrieved using batched REST API queries from two complementary sources: (i) myvariant.info, queried using dbSNP rsIDs (POST batch size = 200 variants) [17], and (ii) Ensembl Variant Effect Predictor (VEP, Ensembl REST API release 113), queried by genomic coordinates (POST batch size = 150 variants) [18]. From myvariant.info, predictor scores were extracted from dbNSFP v4.4, including REVEL, CADD-Phred (CADD v1.7), PolyPhen-2 (HumDiv and HumVar), SIFT (score and rank-score), MutationTaster rank-score, AlphaMissense score and prediction class, and GERP++ RS conservation scores [11,12,13,15,19,20,21]. In parallel, Ensembl VEP was used to retrieve canonical transcript-level annotations, including protein position, amino acid substitutions, codon changes, consequence terms, predicted impact, and exon-level information. VEP-derived SIFT and PolyPhen scores were additionally used to supplement missing dbNSFP values, ensuring maximal annotation completeness. To improve efficiency and reproducibility, all API responses were cached locally, enabling incremental updates and eliminating redundant network queries in subsequent runs. This batching and caching strategy substantially reduced annotation runtime while maintaining consistency across analyses. Per-predictor coverage statistics for the missense training subset are reported in Section 3.2 and informed downstream handling of missing predictors and the implementation of limited-evidence classification tiers.

### 2.3. Splice Annotation for Non-Coding Variants

This section describes how non-coding variants were assessed for potential effects on RNA splicing. Two distinct uses of splice annotation should be distinguished. (i) Splice-related features used in model training: During primary feature generation, a single binary indicator (is_splice_variant_vep) derived from the Ensembl Variant Effect Predictor (VEP) SpliceRegion plugin was created for each non-coding SNV [18]. Specifically, for non-coding single-nucleotide variants (SNVs) belonging to the consequence categories non_coding, 5′-UTR, 3′-UTR, intronic, and unknown (n = 636), variants annotated with splice-related consequence terms in the *LDLR* canonical transcript were classified as splice-involved (is_splice_variant_vep = 1), identifying 200 variants with potential splicing impact. This indicator was retained in the engineered feature matrix used to fit the all-variant XGBoost model. To preserve a clean and reproducible benchmark, the splice indicator was deactivated in the downstream ablation, SHAP, and external validation runs reported in the manuscript; the variant remained in those analyses, but its is_splice_variant_vep value was zeroed so that comparative metrics are not influenced by the binary splice flag. (ii) Splice annotations applied as post hoc prioritization rules: Independently of model training, splice-region annotations were re-used by the VUS prioritization framework (Section 2.7, Table 1) as a rule-based “splice-rescue” step that converts otherwise unresolved non-coding VUS into the dedicated tiers P_LP_splice_evidence (splice + p ≥ 0.5) and B_LB_splice_low (splice + p < 0.5). The rule-based step is therefore strictly a post-processing layer that operates on calibrated XGBoost probabilities; it does not alter the trained model or the predicted probability of any variant. This separation is intended to allow readers and adopters to reproduce or swap each layer independently—for example, replacing the binary VEP-derived flag with continuous SpliceAI scores [22] while keeping the same rule-based rescue tier.

### 2.4. Feature Engineering

This section describes how raw annotations were transformed into the numerical features used as model inputs. The pipeline constructs 45 base features from the annotated dataset, which expands to 68 encoded features after one-hot encoding of categorical variables (Table S1). Features are organized into nine groups: (1) sequence features, including REF/ALT lengths, length difference, SNV/indel indicators, and transition/transversion status; (2) allele frequency features with explicit handling of missingness, including per-source frequencies (ESP, ExAC, 1000 Genomes), measurement flags, maximum and log-transformed AF, and a discretized frequency bin (af_bin ∈ (not_measured, absent_measured, ultra_rare, rare, low_freq, common)); (3) molecular consequence and variant class derived from MC and CLNVC; (4) in silico predictors, including REVEL, CADD-Phred, PolyPhen-2, SIFT (and inverted SIFT), AlphaMissense, and MutationTaster, along with availability indicators and predictor count; (5) a consensus pathogenicity score computed as the mean of normalized predictor scores; (6) amino acid biochemistry features, including Grantham distance, hydrophobicity change, charge difference, and structural flags (e.g., cysteine loss, proline gain); (7) splice indicator; (8) allele origin; and (9) interaction features capturing biologically relevant combinations (e.g., rare missense, rare loss-of-function, REVEL–frequency interactions).

### 2.5. Model Training and Evaluation

**Figure 1:**
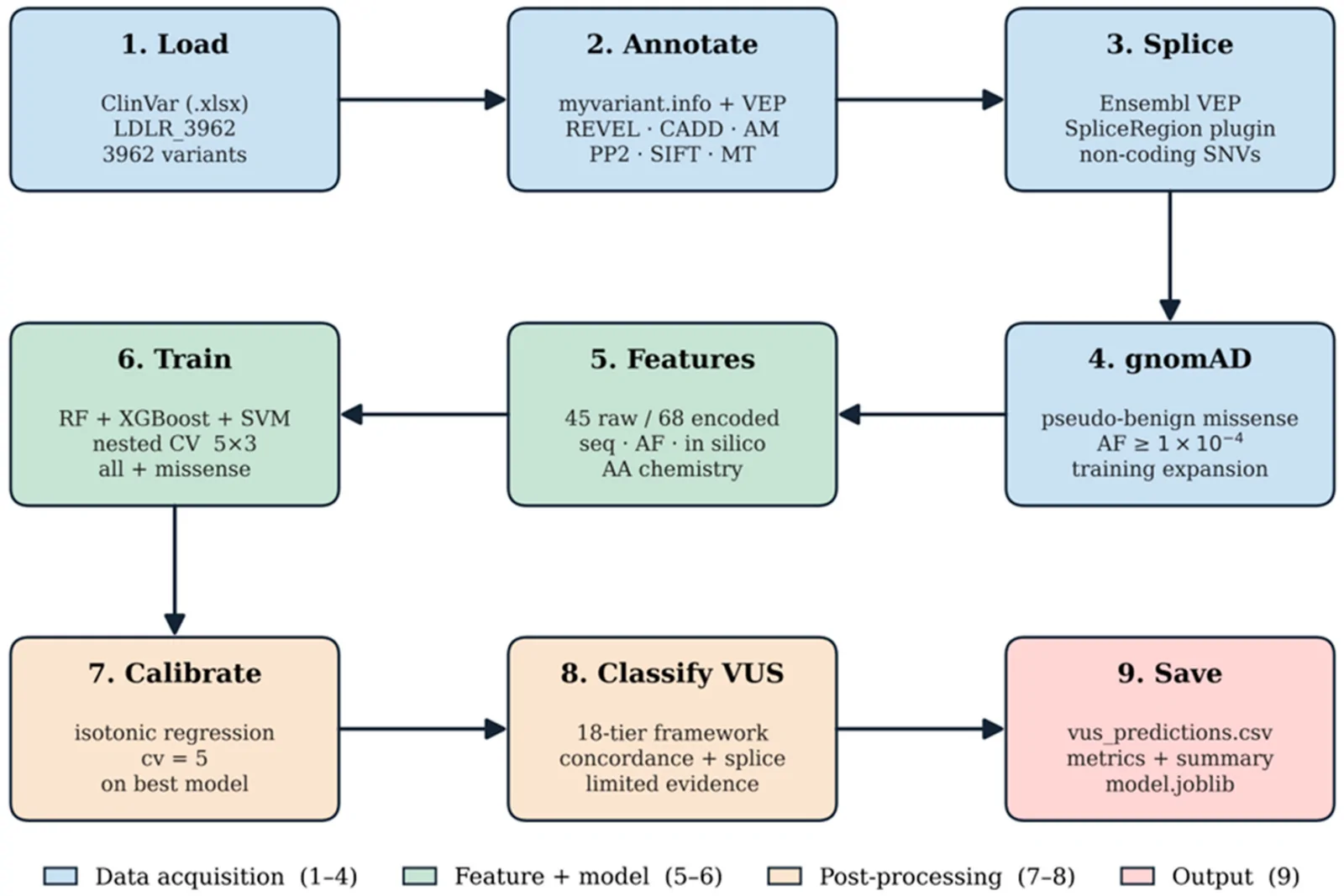
Overview of the *LDLR* VUS classification pipeline. Schematic representation of the end-to-end pipeline for prioritizing variants of uncertain significance (VUS) in the *LDLR* gene. Stages 1–4 (blue) encompass data acquisition and annotation, including ClinVar variant input and integration of external annotations from myvariant.info, Ensembl Variant Effect Predictor (VEP), and gnomAD. Stages 5–6 (green) involve feature engineering (45 raw features expanded to 68 encoded features) and model training using RandomForest, XGBoost, and SVM under nested 5 × 3 cross-validation, with parallel all-variant and missense-only models. Stages 7–8 (tan) perform probability calibration using isotonic regression and classify VUS within a multi-tier framework that incorporates predictor concordance, limited evidence, and splice-rescue rules. Stage 9 (pink) summarizes outputs, including variant-level predictions, performance metrics, and trained models.

This section describes the machine learning algorithms tested and the cross-validation strategy used to assess their performance. Three model classes were evaluated: RandomForest (scikit-learn v1.7.2), XGBoost v3.1.2, and support vector machine (SVM; scikit-learn v1.7.2, RBF and linear kernels with StandardScaler preprocessing, probability = True, and class_weight = “balanced”) [23,24]. All analyses were implemented in Python v3.13.9 using NumPy v2.3.5, pandas v2.3.3, and matplotlib v3.10.7. Models were trained using a nested cross-validation framework (Figure 1), with an outer 5-fold StratifiedKFold for unbiased performance estimation and an inner 3-fold StratifiedKFold with RandomizedSearchCV (20 configurations, ROC-AUC scoring) for hyperparameter tuning. Class imbalance was addressed using class weighting for RandomForest and inverse-frequency sample weights for XGBoost. Missing values were handled by median imputation fitted exclusively on training folds to prevent data leakage. Two complementary models were developed: an all-variant model (Tier A), trained on all 2870 P/LP and B/LB variants to assess overall classification performance across variant types, and a missense-only model (Tier B), trained exclusively on missense variants (P/LP ≈ 732, B/LB = 54 after gnomAD expansion), targeting the clinically critical task of within-missense discrimination. The best-performing all-variant model, selected by mean nested-CV AUROC, was subsequently calibrated using isotonic regression (CalibratedClassifierCV, cv = 5). Final performance metrics, including calibrated AUROC and Brier score, were estimated on a held-out 20% test split.

### 2.6. Predictor Concordance

This section defines how agreement or disagreement between two key predictors (REVEL and AlphaMissense) was quantified for each variant. For each VUS, a predictor_concordance label was derived based on the agreement between REVEL and AlphaMissense [11,12]. Variants were classified as concordant_pathogenic (REVEL ≥ 0.7 and AlphaMissense ≥ 0.564) or concordant_benign (REVEL < 0.3 and AlphaMissense < 0.34) when both predictors agreed in direction. Discordant categories were defined when predictors disagreed, including discordant_REVEL_path_AM_benign (REVEL ≥ 0.5 and AlphaMissense < 0.34) and discordant_AM_path_REVEL_benign (AlphaMissense ≥ 0.564 and REVEL < 0.5). Variants not meeting these criteria were labeled as both_uncertain, while those lacking one or both predictors were assigned to insufficient_predictors. Thresholds were based on established REVEL pathogenicity cut-offs and AlphaMissense classification boundaries [11,12]. This concordance framework was used to explicitly capture conflicting computational evidence and prevent overconfident classification of variants with divergent predictor signals.

### 2.7. VUS Prioritization Framework

This section defines the rule-based tiering system that converts model outputs into actionable confidence categories. VUS were assigned to 18 calibrated confidence tiers (Table 1) using the predicted P_LP probability (p), predictor_concordance, and splice annotation. Splice evidence was applied first for non-coding variants, identifying P_LP_splice_evidence (splice + p ≥ 0.5) and B_LB_splice_low (splice + p < 0.5). Variants with conflicting predictor signals were explicitly flagged as Predictor_Discordant_VUS and excluded from directional classification. The remaining variants were stratified into four confidence levels based on calibrated probability thresholds. High-confidence variants had p ≥ 0.90 or p ≤ 0.10. Moderate-confidence variants fell within 0.80 ≤ p < 0.90 or 0.10 < p ≤ 0.20. Low-confidence variants had 0.65 ≤ p < 0.80 or 0.20 < p ≤ 0.35. Variants with 0.35 < p < 0.65 were classified as unresolved (Table 1). Concordance between REVEL and AlphaMissense was used to further support high-confidence classifications, while variants lacking both predictors were assigned to limited-evidence categories. This framework prioritizes interpretability and uncertainty awareness by explicitly separating concordant, discordant, and insufficient-evidence variants, thereby avoiding overconfident classification.

### 2.8. Feature-Group Ablation and SHAP Interpretation

This section describes (a) how the contribution of individual feature groups was measured by leave-one-group-out ablation of the deployed XGBoost classifier, and (b) how model decisions were interpreted post hoc using SHAP values computed on a RandomForest surrogate. To quantify the contribution of each feature group, the all-variant and missense-only XGBoost models were each refitted after removing one feature group at a time. The seven groups tested were: in silico predictors, allele frequency, amino acid biochemistry, splice indicator, consequence, interaction features, and sequence features. Performance was evaluated using fixed hyperparameters and 5-fold StratifiedKFold, and the change in ROC-AUC relative to the full model was reported as ΔAUC. For model interpretation, Shapley additive explanations (SHAP, SHAP v0.49.1) were computed on a held-out 25% test split using TreeExplainer applied to a RandomForest surrogate trained on the same engineered features [25,26]. We emphasized that the resulting SHAP values describe the surrogate model rather than the deployed XGBoost classifier; TreeExplainer was used on RandomForest because of compatibility limitations between SHAP TreeExplainer and recent XGBoost model serialization formats. The two models share the same training data and feature space but differ in optimization objective and tree structure, so the SHAP-based rankings reported here should be read as an approximation of the global feature-importance structure of the XGBoost model. We therefore use SHAP only to corroborate the qualitative ranking observed in the consequence-aware ablation analysis (Section 3.8) and explicitly caution against over-interpretation of the precise ordering of individual features or of small differences in mean |SHAP| values.

### 2.9. ACMG/AMP PP3/BP4 Evidence-Code Mapping

This section explains how calibrated model outputs were mapped to standardized clinical evidence codes used in variant curation. For each VUS, ACMG/AMP PP3/BP4 computational evidence codes [8] were assigned using Pejaver-calibrated REVEL thresholds [10]: PP3_Strong (≥0.932), PP3_Moderate (≥0.773), PP3_Supporting (≥0.644), BP4_Strong (≤0.016), BP4_Moderate (≤0.183), BP4_Supporting (≤0.290), and Indeterminate otherwise. AlphaMissense scores were mapped to PP3_Supporting (≥0.564) or BP4_Supporting (<0.34) based on published thresholds [12]. When both predictors agreed in direction, the stronger evidence level was retained; discordant predictions were explicitly labeled as “Conflicting,” consistent with the Predictor_Discordant_VUS category. This mapping framework is conceptually grounded in the Bayesian evidence weighting system proposed by Tavtigian et al., linking supporting, moderate, and strong computational evidence to quantitative pathogenicity inference [27,28].

### 2.10. Split-Half Internal Validation

To confirm that model performance is robust and not dependent on the specific training sample, we conducted three complementary split-half analyses. In all cases, the 2870 labeled variants (P_LP/B_LB) were divided into two equal halves using stratified random sampling (maintaining the P_LP:B_LB ratio in each half), and the 1092 VUS were held out for prediction.

*Approach A—Within-partition VUS prediction.* The labeled variants were split into Set 1 and Set 2 (940 P_LP + 495 B_LB each; random seed = 42). A model trained on Set 1 predicted VUS in the same partition and was evaluated on Set 2’s labeled variants (and vice versa). This yielded unbiased performance estimates across all labeled variants while also generating VUS predictions from each half-model.

*Approach B—Cross-partition evaluation.* Each half-model predicted only the opposite labeled set, ensuring every labeled variant was evaluated strictly out-of-sample. Performance was assessed both globally and within the missense subset, providing a direct comparison to full-data cross-validation results.

*Approach C—Multi-seed stability assessment.* The split-half procedure was repeated across five independent random seeds (42–46), generating ten calibrated XGBoost models (two per seed). Each VUS received ten independent probability estimates. From these, we computed: (i) the mean predicted probability, (ii) the standard deviation (SD) as a measure of classification instability, and (iii) the fraction of models predicting pathogenic (vote fraction). Variants were assigned to stability tiers based on SD: stable (SD < 0.05), mildly unstable (0.05–0.10), moderately unstable (0.10–0.20), and highly unstable (≥0.20). A consensus classification was derived from the majority vote across all ten models.

### 2.11. External Validation Against the ClinGen FH-VCEP

This section describes how the model was independently tested against variants curated by an expert panel. To externally validate the pipeline, the all-variant XGBoost model was trained on 2651 P_LP/B_LB variants from ClinVar that were not reviewed by an expert panel (i.e., 1- and 2-star records) and evaluated on 219 variants curated by the ClinGen FH Variant Curation Expert Panel (3-star expert-panel review) [7]. As the expert panel independently reclassifies variants using *LDLR*-specific ACMG/AMP criteria, this setup provides a high-confidence validation with minimal label overlap between training and test sets. Model performance was assessed using ROC-AUC, PR-AUC, F1 score, Brier score, sensitivity, and specificity, both overall and stratified by molecular consequence. In addition, predictions were generated for 275 expert-panel VUS to evaluate classification behavior and tier distribution in an independent uncertain set. To facilitate future benchmarking, the pipeline includes a flexible user-supplied external validation mode, enabling validation on external datasets such as LOVD-LDLR and other curated variant repositories [29].

### 2.12. Reproducibility

This section summarizes the software environment and code availability for reproducing all analyses. The complete analysis was implemented using custom Python (version 3.13.9)-based pipelines, enabling full reproducibility of all results. To ensure determinism, all analyses were conducted with fixed random seeds (SEED = 42). API-based annotations were cached locally, allowing subsequent runs to execute without network access while producing identical results. All source code and scripts are publicly available as described in the Data and Code Availability section. All preprocessing, model fitting, and evaluation steps were executed using version-controlled scripts to maximize reproducibility.

## 3. Results

### 3.1. Cohort

The *LDLR*_3962 dataset derived from ClinVar comprised 3962 variants following CLNSIG normalization, including 1880 P_LP, 990 B_LB, and 1092 VUS (Figure 1; Table 2). The 2870 P_LP and B_LB variants were used for model training, while VUS were reserved as the prediction target. Among the VUS, missense variants predominated (n = 776, 71.1%), followed by intronic (n = 81, 7.4%), non-coding (n = 73, 6.7%), 3′-UTR (n = 70, 6.4%), and 5′-UTR variants (n = 30, 2.7%). Remaining categories were relatively rare, including in-frame deletions (n = 18), synonymous variants (n = 18), in-frame insertions (n = 9), splice donor variants (n = 6), frameshift variants (n = 5), splice acceptor variants (n = 2), unknown (n = 3), and other variant types (n = 1).

### 3.2. Annotation Coverage

Across the full *LDLR* variant set (n = 3962), annotation coverage was high for most in silico predictors, with limited missingness primarily affecting REVEL and AlphaMissense due to database availability constraints. Within the missense training subset (n = 776), coverage was 87.0% for REVEL, 93.2% for CADD, 87.0% for AlphaMissense, and 98.1% for both PolyPhen-2 and SIFT. Protein position and amino acid change annotations were recovered for 96.5% of missense variants. These results indicate that while most predictors provide near-complete coverage, systematic gaps remain for specific tools, particularly REVEL and AlphaMissense. This uneven availability informed the incorporation of limited-evidence labeling and motivated the explicit modeling of predictor concordance, enabling robust handling of missing or conflicting computational evidence.

### 3.3. Model Performance

**Figure 2:**
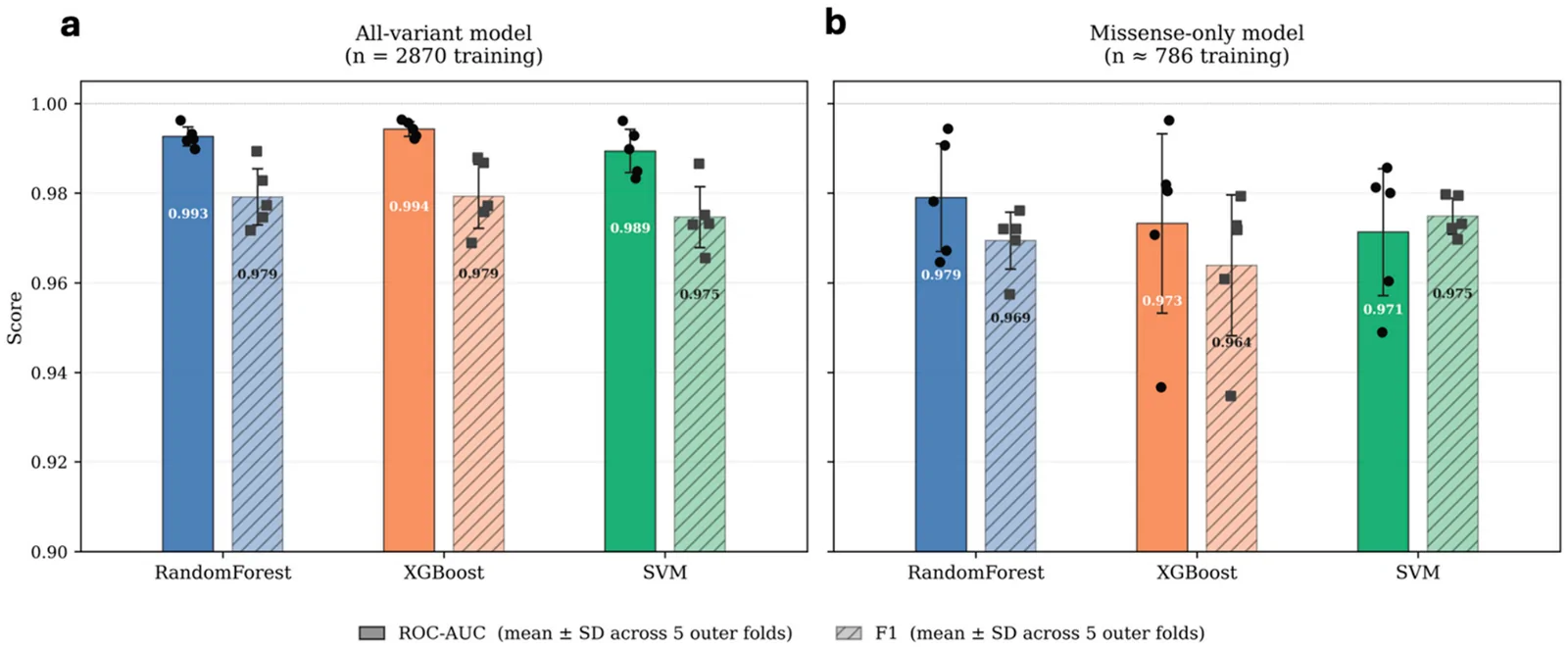
Nested 5 × 3 cross-validation performance of classification models. Performance of RandomForest, XGBoost, and SVM classifiers evaluated using nested 5 × 3 cross-validation. Solid bars represent mean ROC-AUC across the five outer folds, and hatched bars represent mean F1 score; error bars indicate ±1 standard deviation, and overlaid points correspond to individual fold values. (**a**) All-variant model trained on 2870 P_LP/B_LB variants from ClinVar. (**b**) Missense-only model trained on 786 missense variants following gnomAD-based pseudo-benign expansion. Overall, all models achieve near-perfect discrimination in the all-variant setting, whereas performance is modestly reduced in the missense-only setting, reflecting increased classification difficulty within this clinically relevant subset.

Nested cross-validation results for all three model classes across both modeling tiers are summarized in Table 3 and visualized in Figure 2. Across the full variant set, all models demonstrated excellent discrimination, with XGBoost achieving the highest mean ROC-AUC (0.9943 ± 0.0018), followed closely by RandomForest (0.9926 ± 0.0024) and SVM (0.9894 ± 0.0054). Corresponding F1 scores were consistently high (~0.97–0.98), indicating robust classification performance across methods. Performance in the missense-only setting was slightly reduced but remained strong, with ROC-AUC values of 0.9732 ± 0.0223 for XGBoost, 0.9790 ± 0.0134 for RandomForest, and 0.9713 ± 0.0158 for SVM. Notably, all three model classes converged to a similar performance range (AUC ~0.97–0.98), suggesting that predictive performance in this setting is primarily constrained by the underlying signal captured by in silico predictors rather than by model-specific capacity or overfitting.

**Figure 3:**
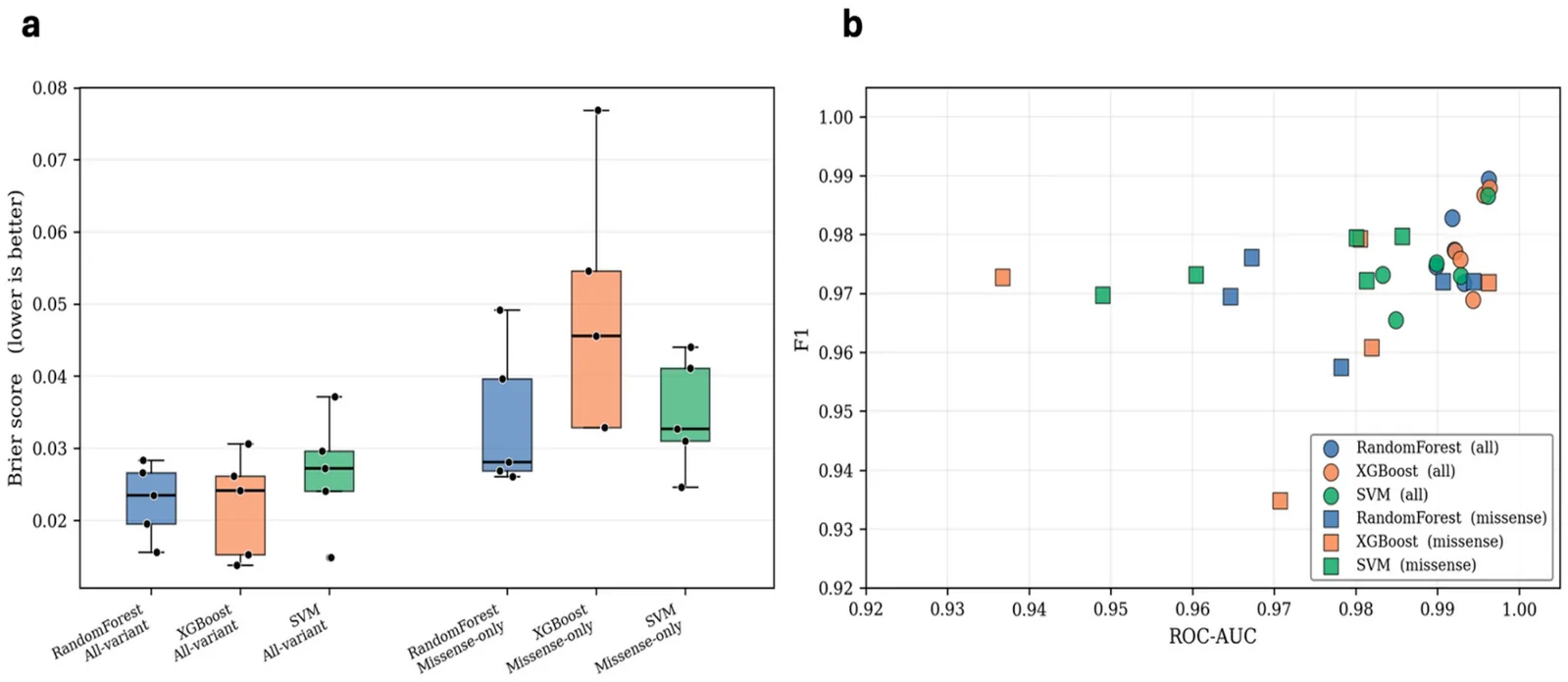
Calibration and per-fold performance relationships. (**a**) Distribution of per-fold Brier scores for RandomForest, XGBoost, and SVM models across both all-variant and missense-only tiers. Lower Brier scores indicate better calibration of predicted probabilities. (**b**) Relationship between per-fold ROC-AUC and F1 scores. Circles denote all-variant models and squares denote missense-only models. Across folds, ROC-AUC and F1 exhibit a consistent positive relationship, indicating stable model performance with respect to both discrimination and class balance. The relatively low and tightly distributed Brier scores further demonstrate reliable probability calibration across model types and variant tiers.

The missense-only models exhibited higher fold-to-fold variability compared to the all-variant models, consistent with the relatively small number of benign missense variants available for training. Despite this, discrimination remained consistently high, indicating stable learning of within-missense patterns. Calibration performance, assessed using Brier scores, is shown in Figure 3 and demonstrated good agreement between predicted probabilities and observed outcomes across all models. Importantly, the missense-only AUROC of approximately 0.97 substantially exceeds trivial within-missense baselines, confirming that the models capture meaningful discriminatory signals from in silico predictors and amino acid biochemistry features rather than relying on variant consequence alone [16].

### 3.4. VUS Prioritization

The calibrated all-variant model generated P(pathogenic) probabilities for each of the 1092 VUS, which were subsequently mapped to confidence tiers as defined in Section 2.7 (Table 1).

**Figure 4:**
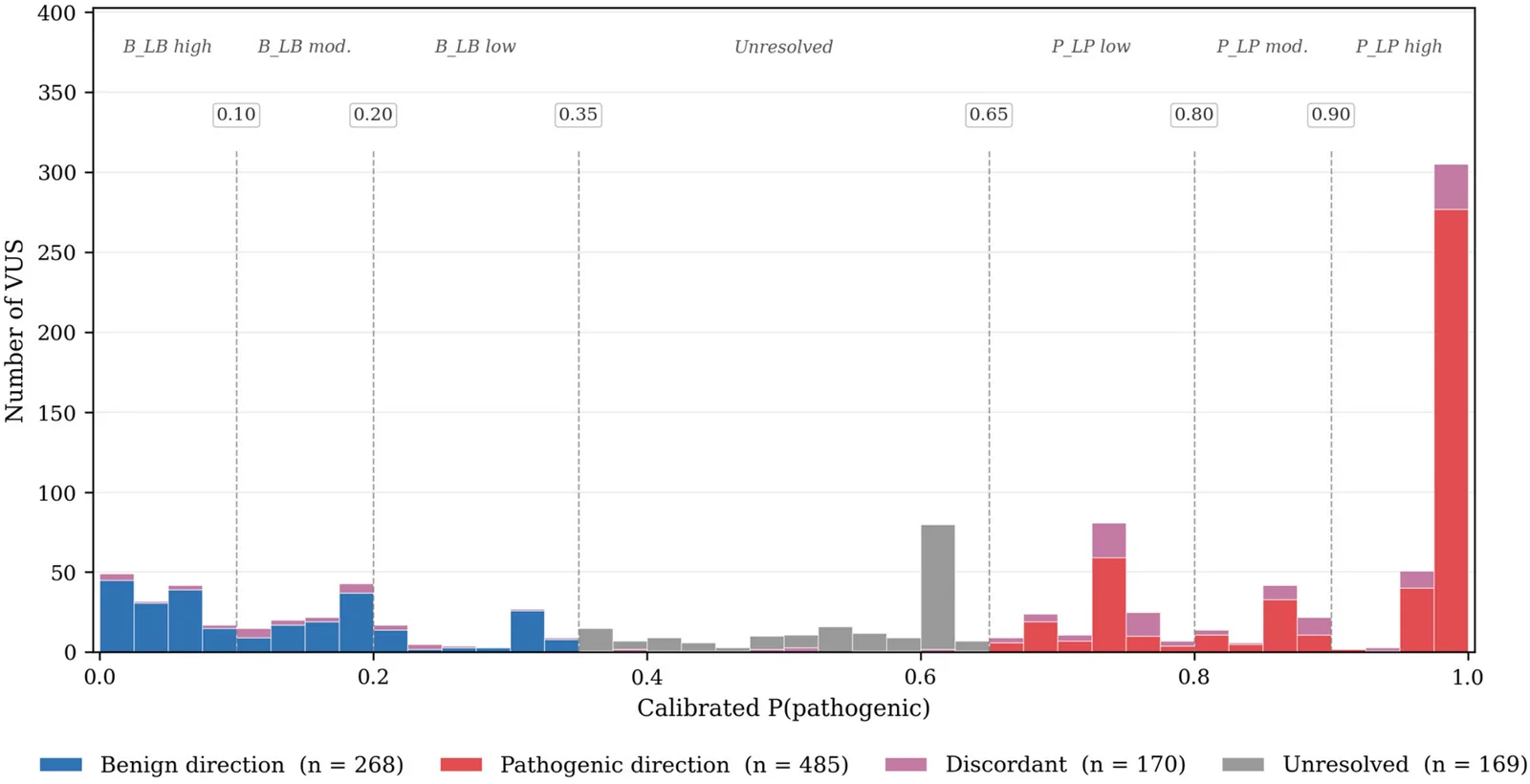
Distribution of calibrated pathogenic probabilities across *LDLR* VUS. Histogram of calibrated P(pathogenic) for 1092 *LDLR* VUS, stratified by final classification direction: benign (blue), pathogenic (red), discordant (purple), and unresolved (gray). Vertical dashed lines indicate classification thresholds (0.10, 0.20, 0.35, 0.65, 0.80, 0.90), with corresponding confidence tiers annotated above the plot. The distribution exhibits clear bimodality, with enrichment at low and high probability extremes corresponding to high-confidence benign and pathogenic classifications. Variants in the intermediate probability range (0.35–0.65) are designated as unresolved, while the centrally distributed discordant variants represent cases with conflicting predictor evidence that are intentionally not assigned a direction.

The distribution of calibrated probabilities exhibited a clear bimodal pattern, supporting the separation of variants into pathogenic- and benign-leaning groups. Overall, 485 VUS (44.4%) were classified as pathogenic-leaning and 268 (24.5%) as benign-leaning, together accounting for 69.0% of all VUS (Figure 4; Table 4). The remaining variants were explicitly categorized as predictor-discordant (n = 170, 15.6%) or unresolved (n = 169, 15.5%), reflecting either conflicting in silico evidence or intermediate probability values within the abstention range (0.35 < p < 0.65). This distribution highlights the utility of the framework in prioritizing a substantial proportion of VUS while preserving uncertainty through dedicated discordant and unresolved categories, thereby avoiding overconfident classification.

### 3.5. Predictor Concordance

**Figure 5:**
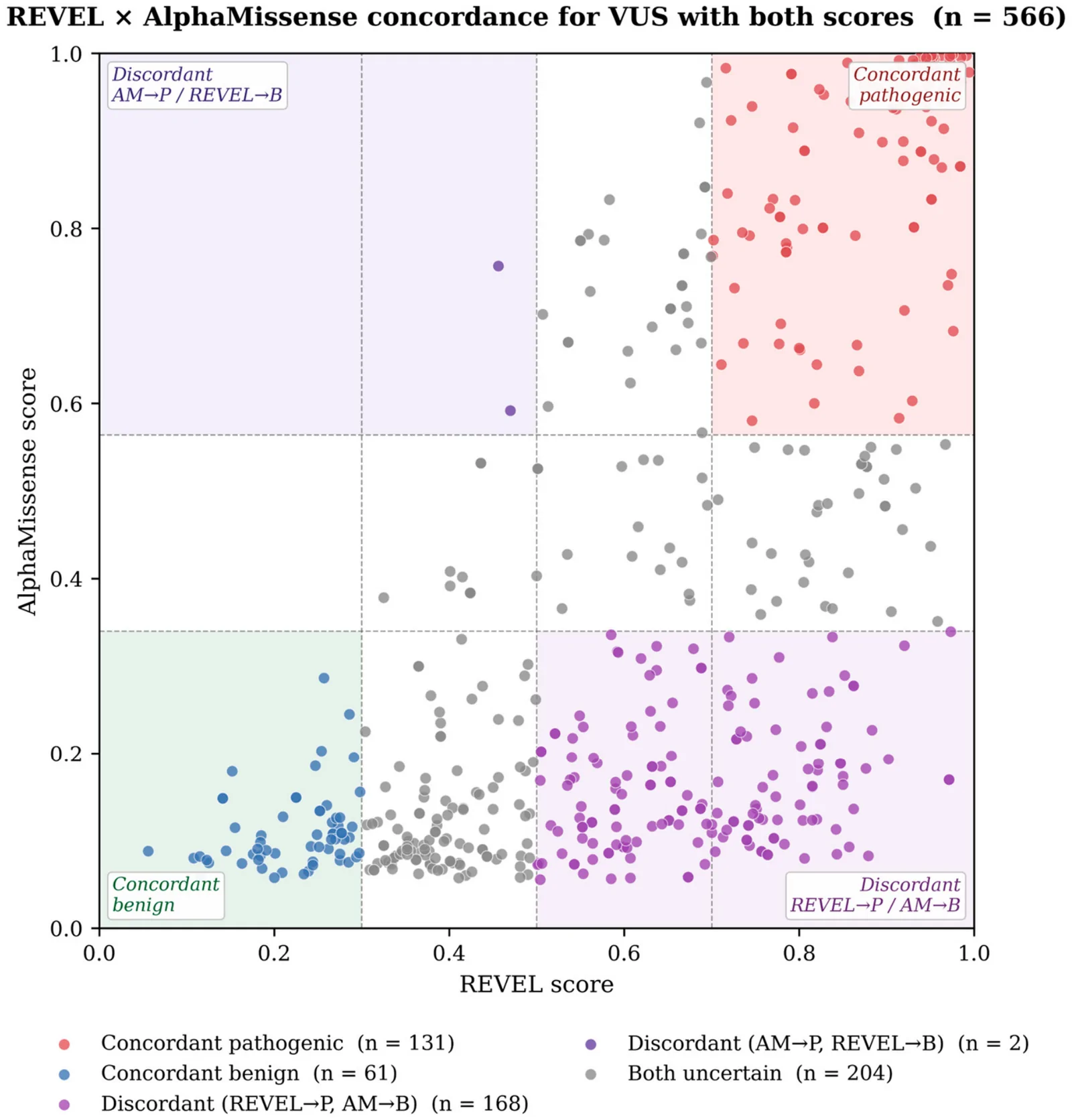
REVEL × AlphaMissense concordance landscape for *LDLR* VUS. Joint distribution of REVEL and AlphaMissense scores for 566 VUS with both predictors available. Shaded regions define concordance and discordance categories based on predefined thresholds: concordant pathogenic (top-right), concordant benign (bottom-left), and two discordant quadrants (top-left and bottom-right). Points are colored by predictor concordance category. The majority of discordant variants cluster in the bottom-right quadrant (REVEL pathogenic/AlphaMissense benign; n = 168), whereas only two variants occupy the opposite discordant quadrant. Variants in the central region represent cases with uncertain or intermediate scores from both predictors. This asymmetric discordance pattern highlights systematic differences between predictor outputs and supports the need for explicit concordance-aware classification.

REVEL and AlphaMissense scores were jointly available for 566 of the 1092 VUS, with their joint distribution shown in Figure 5 and categorical breakdown in Table 5. Among all VUS, 131 variants (12.0%) were classified as concordant pathogenic and 61 (5.6%) as concordant benign. In contrast, 170 variants (15.6%) exhibited clear discordance between predictors. Notably, the discordant set was overwhelmingly dominated by cases where REVEL predicted pathogenicity while AlphaMissense indicated benign effect (168 of 170 variants), consistent with previously reported differences in predictor training paradigms and feature representations rather than true biological disagreement [10,16]. Within the proposed framework, these variants were not assigned directional classification but were instead explicitly flagged as Predictor_Discordant_VUS, prioritizing them for orthogonal evidence evaluation. Importantly, under simpler aggregation strategies (e.g., mean-score or majority voting), these discordant variants would have been disproportionately assigned to high-confidence pathogenic tiers, potentially inflating false-positive classifications. The explicit modeling of predictor discordance therefore represents a critical safeguard against overconfident interpretation. A substantial proportion of variants (n = 526, 48.2%) lacked one or both predictor scores and were categorized as insufficient_predictors, while 204 variants (18.7%) fell into the both_uncertain category. These observations further support the need for uncertainty-aware classification strategies when integrating heterogeneous in silico evidence.

### 3.6. Missense Subset

**Figure 6:**
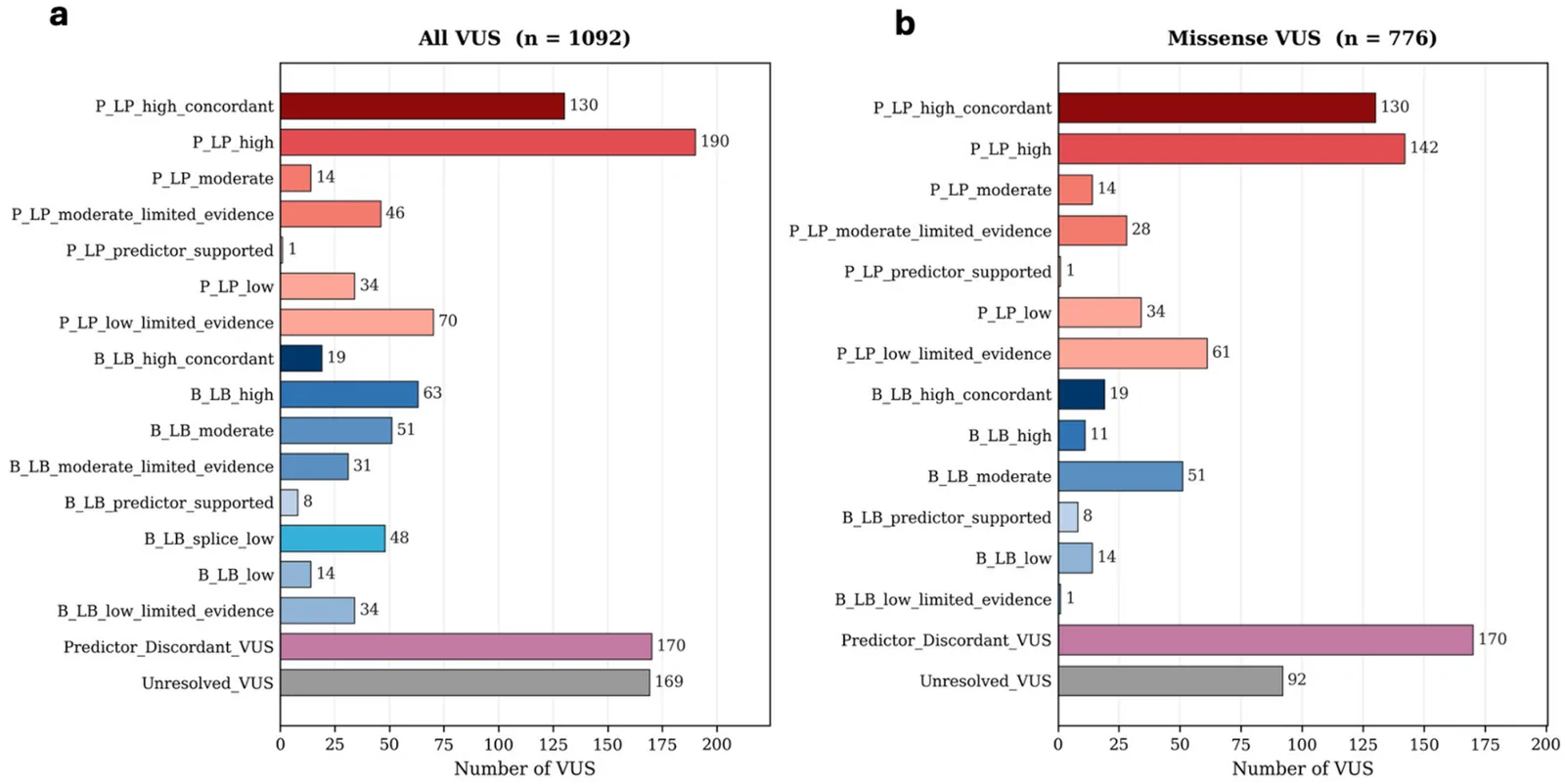
Distribution of VUS across classification tiers. Bar plots show the distribution of variants of uncertain significance (VUS) across classification tiers. (**a**) All VUS (n = 1092). (**b**) Missense-only subset (n = 776). Bars are colored by classification direction: pathogenic (red), benign (blue), discordant (purple), unresolved (gray), and splice-rescued benign (light blue). Color intensity reflects confidence level, ranging from high-concordance (darkest) to limited-evidence categories (lightest). Across both panels, a substantial proportion of variants are assigned to high-confidence pathogenic and benign tiers. In the missense subset, the largest category is Predictor_Discordant_VUS, highlighting the prevalence of conflicting in silico evidence within clinically relevant variants and underscoring the importance of explicit concordance-aware classification.

Within the clinically critical subset of 776 missense VUS, the tier distribution is shown in the right-hand panel of Figure 6. The largest category was Predictor_Discordant_VUS (n = 170), accounting for approximately 22% of missense variants. The prominence of this discordant group highlights a key finding of the present study: a substantial fraction of *LDLR* missense VUS exhibits conflicting in silico predictions and cannot be reliably assigned a direction based on consensus scoring alone. Under conventional aggregation strategies (e.g., mean-score or majority voting), many of these variants would be assigned to high-confidence pathogenic tiers, potentially leading to systematic overclassification. By explicitly isolating discordant variants, the proposed framework preserves uncertainty and prioritizes these cases for orthogonal evidence, such as functional assays or segregation data. This result underscores the importance of incorporating predictor concordance into variant prioritization, particularly within missense-dominated disease contexts.

### 3.7. Splice Rescue

Of the 636 non-coding SNVs annotated using Ensembl Variant Effect Predictor (VEP) with the SpliceRegion plugin, 200 were identified as splice-involved based on *LDLR* canonical transcript annotations [18]. Among these, 48 VUS were not confidently classified by the all-variant model using standard probability thresholds. Application of the splice-rescue rule reclassified these variants into the B_LB_splice_low category, thereby reducing the size of the unresolved tier. This result demonstrates the utility of incorporating splice-aware annotations to improve classification coverage for non-coding variants that would otherwise remain uninformative under sequence- and predictor-based models alone.

### 3.8. Feature-Group Ablation

To quantify the contribution of each feature group, we performed systematic ablation by removing one group at a time and refitting the all-variant and missense-only XGBoost models using fixed hyperparameters and 5-fold StratifiedKFold evaluation. Results are summarized in Table 6. The dominant finding was that removal of the in silico predictor group led to a marked reduction in missense-only performance, with ROC-AUC decreasing from 0.9589 to 0.8046 (ΔAUC = +0.1543). No other feature group altered missense performance by more than 0.005 AUC, indicating that within-missense discrimination is almost entirely driven by in silico predictors and their derived features. In contrast, removal of the consequence feature group resulted in a substantial drop in all-variant performance (ΔAUC ≈ +0.0215) while having negligible impact on missense-only AUC. This divergence confirms that the all-variant metric is strongly influenced by consequence-based signals, whereas the missense-only metric provides a more accurate assessment of true discriminative capacity within variant classes. Together, these results demonstrate that model performance in the missense setting reflects genuine predictive signals from functional annotations rather than trivial consequence-based shortcuts, supporting the use of missense-restricted evaluation as a more reliable benchmark for variant classification performance [10,16].

### 3.9. SHAP Interpretation

**Figure 7:**
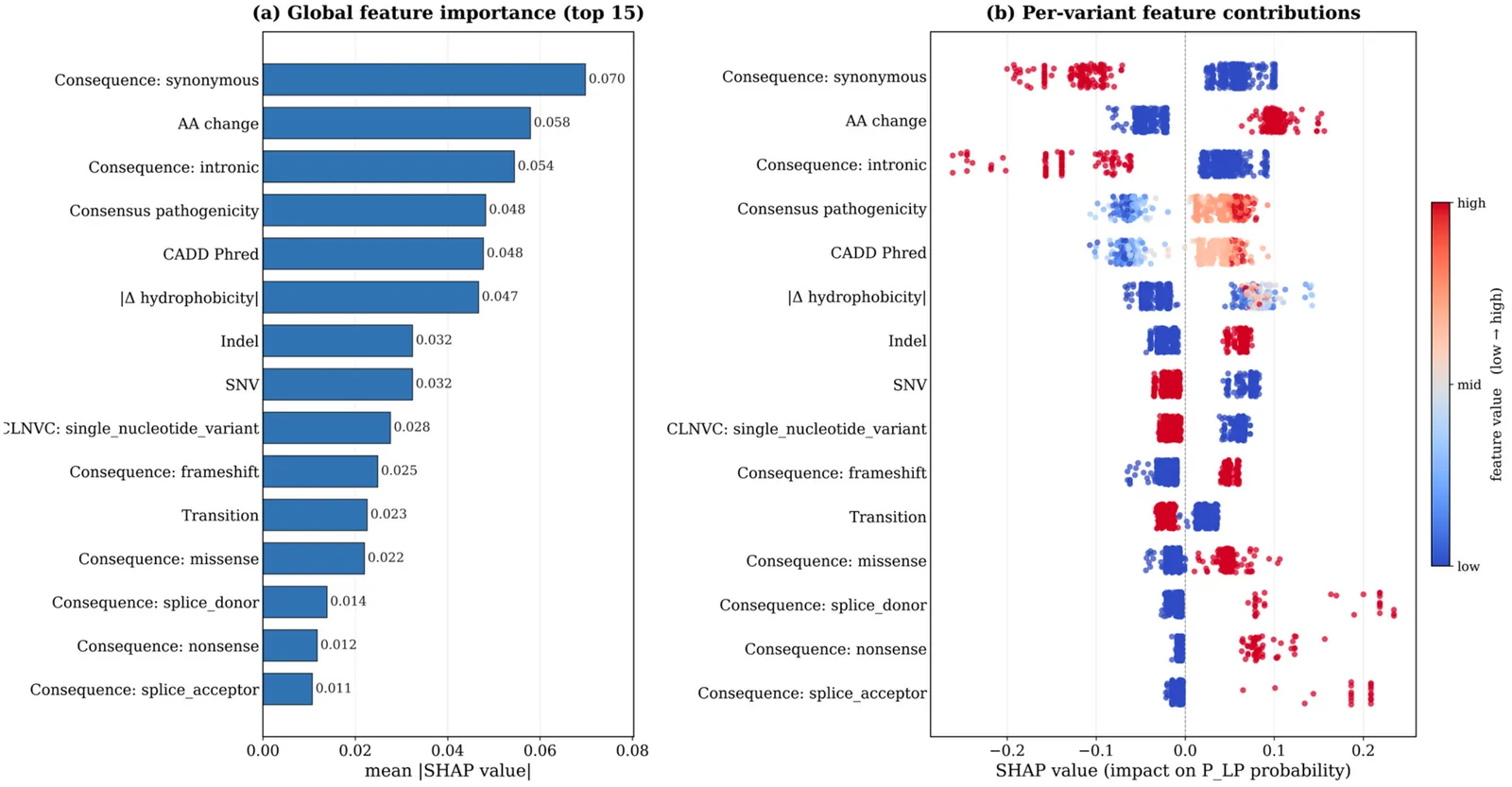
SHAP-based interpretation of feature importance for the all-variant model. Shapley additive explanations (SHAP) were computed with TreeExplainer on a held-out 25% test set (n = 718) using RandomForest classifier. (**a**) Global feature importance showing the top 15 features ranked by mean absolute SHAP value. (**b**) Per-variant SHAP values indicating the contribution of each feature to the predicted probability of pathogenicity (P_LP), with color representing feature value (blue = low, red = high). Feature importance is dominated by molecular consequence indicators (e.g., synonymous, intronic, missense, splice-related) and the consensus pathogenicity score, with CADD and absolute hydrophobicity change among the top contributors. The directionality of effects is biologically consistent: higher consensus pathogenicity scores, elevated CADD values, and greater physicochemical changes increase pathogenic probability, whereas synonymous and intronic features are associated with benign predictions.

Shapley additive explanations (SHAP), computed on a held-out 25% test split using a RandomForest surrogate (Section 2.8), were qualitatively consistent with the feature-group ablation results [25,26]. Because SHAP values are derived from the surrogate rather than the deployed XGBoost model, we interpret the rankings as an approximate description of global feature importance and refrain from drawing conclusions about exact orderings or fine-grained differences between adjacent features. Feature importance, ranked by mean absolute SHAP values, was dominated by molecular consequence indicators (e.g., synonymous, intronic, frameshift, missense, splice donor/acceptor, and nonsense variants) alongside the consensus pathogenicity score. Additional high-ranking features included CADD-Phred and absolute hydrophobicity change (|Δ hydrophobicity|) (Figure 7). The directionality of feature effects (Figure 7b) aligned with biological expectations: higher consensus pathogenicity scores, elevated CADD values, and larger amino acid physicochemical changes were associated with increased pathogenic probability, whereas synonymous and intronic features contributed toward benign predictions. These findings reinforce that the model integrates both functional consequence and biophysical signals to drive classification decisions.

### 3.10. ACMG/AMP PP3/BP4 Evidence-Code Mapping

Mapping model outputs to the Pejaver-calibrated PP3/BP4 framework yielded a spectrum of computational evidence strengths across the 1092 VUS (Table 7) [10]. The most frequent assignments were BP4_Supporting (n = 238, 21.8%), followed by PP3_Moderate (n = 84, 7.7%) and PP3_Strong (n = 62, 5.7%), with an additional 60 variants (5.5%) classified as PP3_Supporting. A total of 99 variants (9.1%) were labeled as Conflicting under the combined REVEL × AlphaMissense rule. The Conflicting category showed strong overlap with the Predictor_Discordant_VUS tier, providing an independent validation of the concordance-based classification strategy. Notably, a substantial proportion of variants (n = 514, 47.1%) lacked sufficient predictor coverage and were assigned as NA, consistent with the limited-evidence categories described earlier. These calibrated evidence codes are directly compatible with ACMG/AMP variant interpretation frameworks and can be integrated into rule-based classification systems, including the Bayesian point-based approach proposed by Tavtigian et al. [27,28]. This enables seamless translation of model outputs into clinically interpretable evidence tiers. Detailed variant-level predictions are provided in Table S2, and summary distributions are shown in Table S3.

### 3.11. Split-Half Internal Validation

Nested 5-fold cross-validation on each labeled half yielded performance essentially identical to the full-data pipeline: Set 1 ROC-AUC 0.9911 (F1 0.976, Brier 0.025) and Set 2 ROC-AUC 0.9934 (F1 0.979, Brier 0.023), compared with 0.9943 for the full-data XGBoost model (Table 3). Applied to the 1092 VUS, the split-half models produced a classification distribution that closely tracked the full-data pipeline (493 P_LP_high, 137 P_LP_moderate, 177 B_LB_high, 134 B_LB_moderate, and 151 uncertain; Table S4, Figure S1), confirming that VUS predictions do not require the full 2870-variant training set. Cross-prediction between the two halves produced fully out-of-sample metrics on all 2870 labeled variants (Table S5, Figure S2), with ROC-AUC 0.9919 (F1 0.975) for Set2→Set1 and 0.9937 (F1 0.982) for Set1→Set2. When restricted to missense variants, ROC-AUC was 0.9532 (n = 373) and 0.9568 (n = 403), slightly below the nested-CV missense AUC of 0.9732, reflecting modest variance in smaller training subsets but remaining well above trivial baselines. Overall, 2789 of 2870 labeled variants (97.2%) were correctly classified at the 0.5 probability threshold, with 1825 true positives, 964 true negatives, 26 false positives, and 55 false negatives. Across five independent 50/50 stratified splits (seeds 42–46), ten calibrated XGBoost models achieved a mean held-out ROC-AUC of 0.9921 (range 0.9879–0.9957; Figure S3), confirming stability across training partitions. Each of the 1092 VUS was scored by all ten models, yielding low variability (mean SD 0.089, median 0.074; Figure S3), with a bimodal stability distribution in which 627 variants (57.4%) were highly stable and 144 (13.2%) were unstable (Table S6, Figure S3). Consensus voting (Table S7, Figure S3) showed that 527 VUS (48.3%) received unanimous pathogenic calls and 199 (18.2%) unanimous benign calls, with an additional 122 (11.2%) achieving near-unanimous agreement, resulting in 848 variants (77.7%) with strong consensus. Notably, model instability was partially independent of the REVEL × AlphaMissense predictor concordance flag, defining an orthogonal uncertainty dimension; variants flagged by either layer represent high-priority candidates for functional validation. A summary comparison of the three internal validation strategies is provided in Table S9.

### 3.12. External Validation Against the ClinGen FH-VCEP

**Figure 8:**
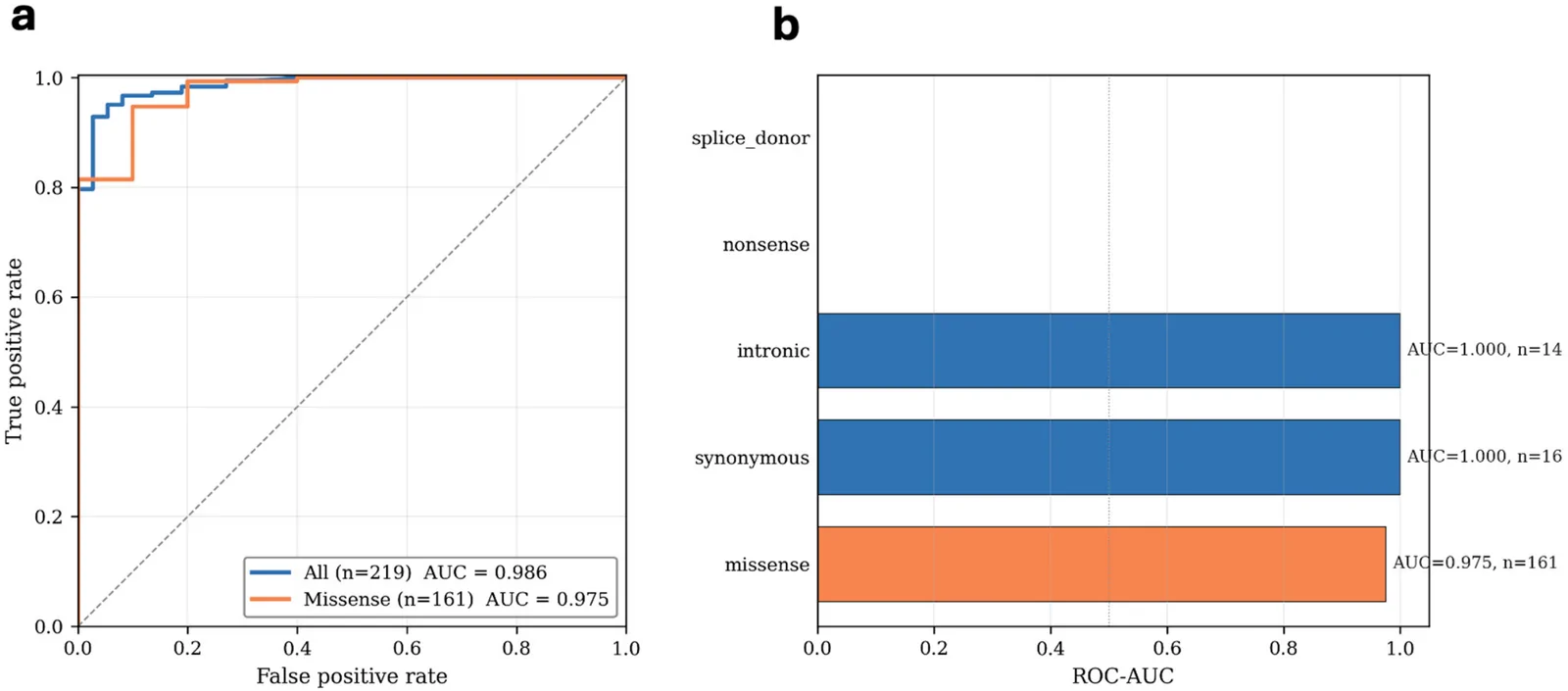
External validation against the ClinGen FH-VCEP expert panel. External validation of the all-variant XGBoost model using an independent expert-curated dataset. The model was trained on 2651 P_LP/B_LB variants from ClinVar that were not reviewed by an expert panel and evaluated on 219 variants curated by the ClinGen FH Variant Curation Expert Panel. (**a**) Receiver operating characteristic (ROC) curves for the full validation set (n = 219) and the missense subset (n = 161), with corresponding AUC values indicated in the legend. (**b**) ROC-AUC stratified by molecular consequence. The missense subset represents the clinically most relevant category, while synonymous and intronic variants achieve near-perfect discrimination. Categories containing only a single class (e.g., nonsense, splice donor) are shown without AUC values, as the metric is undefined. Overall, the model demonstrates strong generalization performance, particularly within the missense subset.

To assess generalizability, the all-variant XGBoost model was retrained on 2651 P_LP/B_LB variants from ClinVar that were not reviewed by the ClinGen FH Variant Curation Expert Panel and evaluated on 219 expert panel-reviewed variants (Table 8, Figure 8). The FH-VCEP provides independently curated classifications using *LDLR*-specific ACMG/AMP criteria, serving as an external gold-standard benchmark [7]. On the held-out FH-VCEP set, the model achieved strong performance (ROC-AUC 0.9861, F1 0.9602, Brier score 0.0568), with high precision (0.994) and sensitivity (0.929) at the default probability threshold (0.5) (Figure 8a). These metrics correspond to 169 true positives, 36 true negatives, one false positive, and 13 false negatives, yielding 205 correct classifications out of 219 variants. Stratified analysis showed robust performance in the clinically relevant missense subset (ROC-AUC 0.9748; n = 161), while near-perfect discrimination was observed for non-missense variants, including synonymous and intronic categories (Figure 8b; Table S8), consistent with their well-defined benign classification. Error analysis identified a small number of misclassifications, primarily false negatives, representing a focused subset for targeted review. Overall, these results demonstrate that the model maintains high discriminative performance when evaluated against independently curated expert-panel data, supporting its robustness and clinical relevance.

### 3.13. Comparative Performance Against Individual Predictors

**Figure 9:**
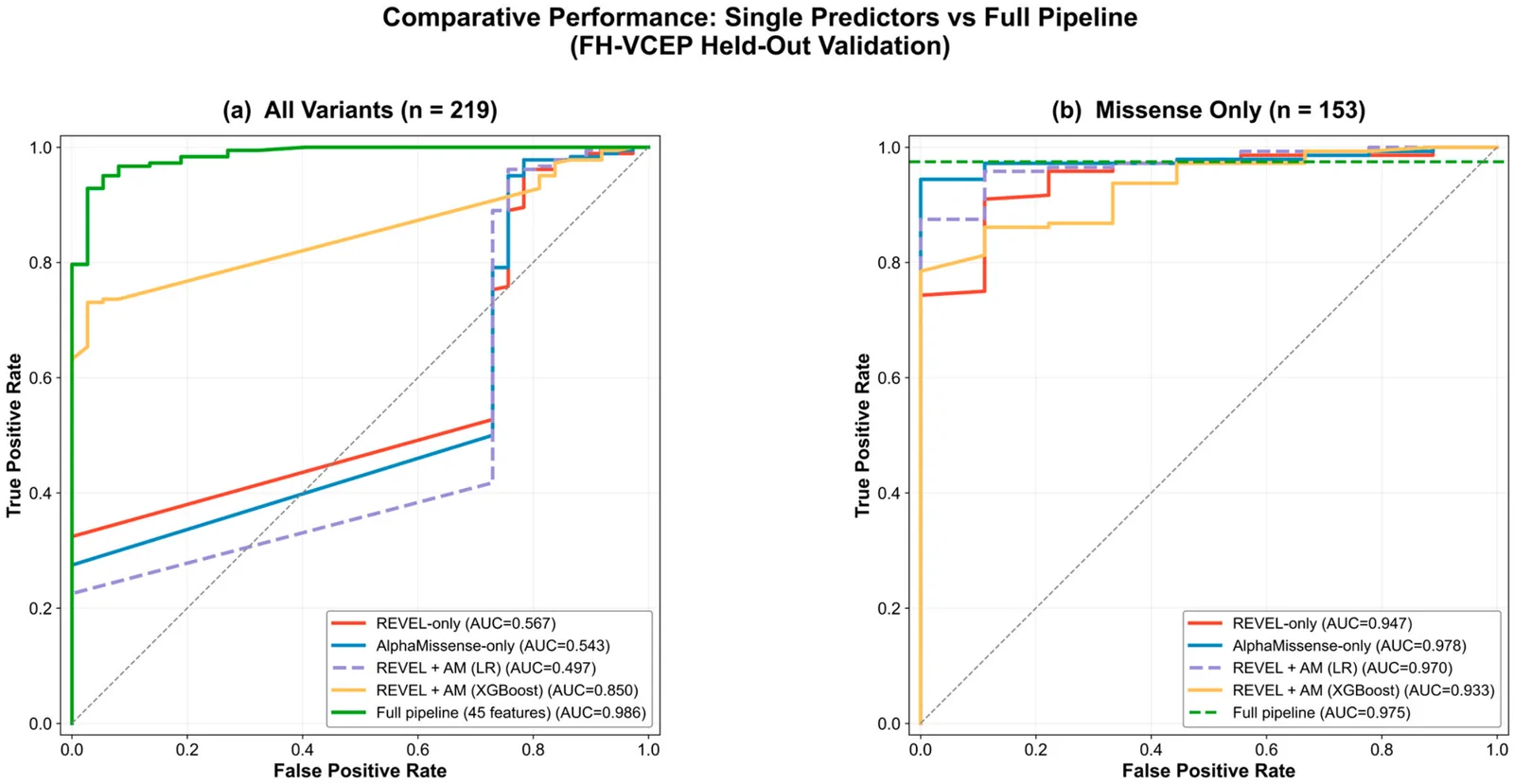
Comparative ROC curves: Single predictors versus full pipeline on FH-VCEP held-out validation. (**a**) All variants (n = 219): REVEL-only and AlphaMissense-only achieve poor discrimination (AUC 0.54–0.57) because they cannot score non-missense variants; the full 45-feature pipeline (AUC = 0.986) substantially outperforms all reduced models. (**b**) Missense-only subset (n = 153): Individual predictors achieve high AUC (0.95–0.98), but the full pipeline maintains superior probability calibration (lowest Brier score = 0.046).

To contextualize the added value of the multi-feature pipeline relative to established individual predictors, we compared the full 45-feature calibrated XGBoost model against: (i) REVEL alone, (ii) AlphaMissense alone, and (iii) the combined use of REVEL and AlphaMissense. All models were evaluated on the same 219-variant ClinGen FH-VCEP held-out validation set (Table 9; Figure 9). In the heterogeneous all-variant test set, individual predictors showed limited discrimination (ROC-AUC 0.54–0.57), largely because REVEL and AlphaMissense are primarily designed for missense variants and do not provide informative scores for approximately 30% of the validation set comprising non-missense variants, including nonsense, frameshift, and splice-site variants. Even the two-predictor XGBoost model based on REVEL and AlphaMissense achieved a lower AUC than the full pipeline (0.850 vs. 0.986), demonstrating the added value of consequence-type features, splice annotations, conservation metrics such as GERP++ and CADD [13], and protein-structural predictors such as PolyPhen-2 and SIFT for classification across the full *LDLR* variant spectrum. These findings are consistent with prior evaluations showing that individual missense predictors can perform well within missense-only analyses but have limited utility across heterogeneous variant classes [16,30].

In the clinically relevant missense-only subset (n = 153), individual predictors achieved high discrimination, with ROC-AUC values of 0.947 for REVEL and 0.978 for AlphaMissense, confirming their established utility for missense variant classification [10,11,12]. However, the full pipeline achieved the lowest Brier score (0.046 vs. 0.146–0.159), indicating superior probability calibration. This is an important advantage because calibrated probabilities are required for mapping continuous model outputs to ACMG/AMP PP3/BP4 evidence-strength tiers, as recommended by Pejaver et al. [10]. Thus, the full pipeline provides two major advantages: first, it can evaluate the complete *LDLR* variant spectrum, including non-coding and splice-region variants that missense-specific predictors cannot adequately score; second, it generates better-calibrated probability outputs that can be translated into graded computational evidence for clinical interpretation.

### 3.14. Clinical Decision-Support Workflow for LDLR VUS Triage

**Figure 10:**
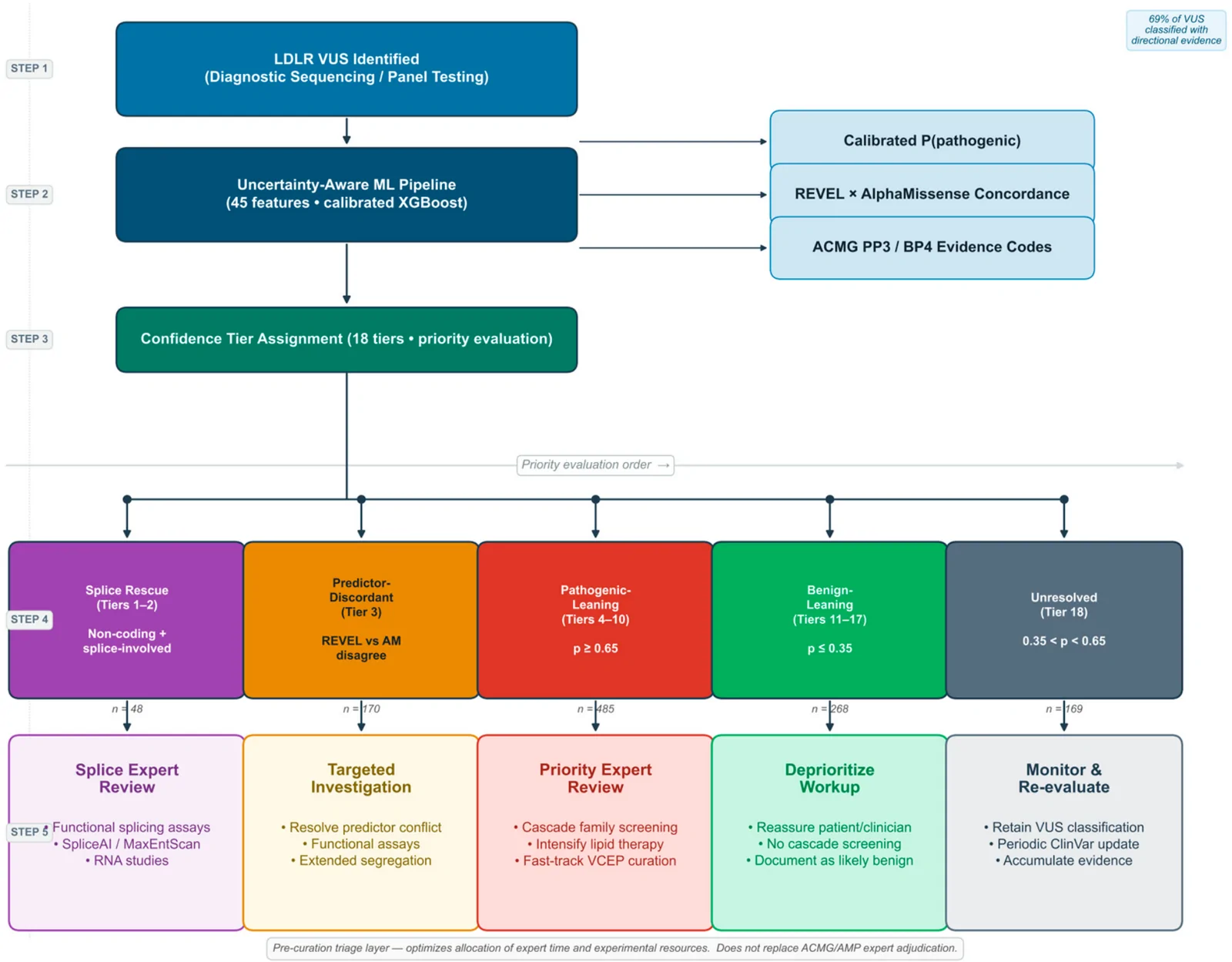
Proposed clinical decision-support workflow for *LDLR* VUS triage. Schematic illustrating how the uncertainty-aware framework integrates into familial hypercholesterolemia (FH) diagnostic workflows. Following detection of an *LDLR* VUS (Step 1), the pipeline generates calibrated probabilities, predictor concordance status, and ACMG/AMP PP3/BP4 evidence codes (Step 2). Based on the assigned confidence tier (Step 3), variants are routed to distinct clinical actions (Step 4): high-confidence pathogenic-leaning variants (Tier 1) are prioritized for expert panel review and cascade screening; high-confidence benign-leaning variants (Tier 5) support deprioritization; predictor-discordant variants (Tier D) are flagged for functional assays; and unresolved variants remain as VUS [7,8].

To illustrate the clinical translation of the proposed framework, Figure 10 presents a decision-support workflow for *LDLR* VUS triage. Following detection of an *LDLR* VUS through diagnostic sequencing or panel testing, the pipeline integrates calibrated pathogenicity probabilities, REVEL–AlphaMissense concordance, and ACMG/AMP-aligned PP3/BP4 evidence codes to assign variants to four categories: pathogenic-leaning, benign-leaning, predictor-discordant, and unresolved.

Pathogenic-leaning variants are prioritized for expert review, segregation assessment, cascade family screening, lipid-management reassessment, and potential fast-track curation. Benign-leaning variants may be deprioritized from intensive workup while remaining documented for formal adjudication. Predictor-discordant variants are not forced into a directional classification and are instead flagged for orthogonal evidence generation, including *LDLR* functional assays, splicing studies, extended segregation analysis, or phenotype–genotype review. Unresolved variants remain classified as VUS and require periodic reassessment as additional clinical, functional, population, or family data become available. Thus, the workflow positions the classifier as a pre-curation triage layer that supports, but does not replace, ACMG/AMP expert adjudication. By organizing computational outputs into transparent and uncertainty-aware tiers, the framework can help prioritize variants for review and optimize the use of clinical and experimental resources.

## 4. Discussion

We developed an uncertainty-aware framework to prioritize *LDLR* variants of uncertain significance (VUS) by integrating multi-tool annotation, calibrated machine learning probabilities, and ACMG/AMP-compatible PP3/BP4 evidence codes. In our ClinVar-derived cohort (3962 *LDLR* variants; 2870 P/LP and B/LB for training; 1092 VUS held out for prioritization), we achieved a practical balance between yield and caution: 69% of VUS received a directional prioritization (44.4% pathogenic-leaning; 24.5% benign-leaning), while 31% were explicitly retained as either predictor-discordant (15.6%) or unresolved (15.5%). This explicit “do not overcall” design becomes most salient in missense VUS, where discordance reached ~22%, a clinically meaningful subgroup that would be silently absorbed by simple consensus approaches.

Our modeling results reinforce that reported discrimination must be interpreted alongside the task definition. On the heterogeneous all-variant task, models achieved ROC-AUC ≈ 0.99, but feature-group ablation and SHAP interpretation indicated that this near-ceiling performance is partly consequence-driven. Specifically, removing consequence one-hot indicators reduced all-variant AUC by ~0.022, consistent with the model exploiting broad, readily separable clinically differences among variant classes. This is concordant with the wider benchmarking literature warning that circularity and dataset composition can inflate observed performance when predictors learn shortcuts rather than within-class biology [31].

In contrast, missense-only evaluation is the clinically faithful benchmark for *LDLR* VUS because missense dominates the held-out VUS pool (71.1%). On this stringent task, three model families converged to ROC-AUC ~0.97–0.98, suggesting that performance is bounded primarily by the information content of available features rather than by classifier choice [16]. Ablation quantified where that information lives: removing the in silico predictor block (including REVEL, CADD, PolyPhen-2, SIFT, AlphaMissense and derived features) collapsed missense ROC-AUC from 0.959 to 0.805 (ΔAUC 0.154), whereas removal of other feature groups changed missense AUC by <0.005. SHAP values—computed using the Shapley additive explanation framework—were consistent with ablation analysis: global feature importance in the all-variant model was dominated by consequence features and a consensus pathogenicity signal, whereas in the missense-only model, predictor scores and amino acid chemistry features (e.g., hydrophobicity change) contributed meaningfully.

The ACMG/AMP guidelines position PP3/BP4 as computational evidence to be used when multiple in silico methods support a consistent direction [8]. However, large-scale evaluation work shows that algorithm concordance is strongly gene- and dataset-dependent, undermining simplistic “multiple tools agree” rules and complicating consistent application of PP3/BP4 [30]. Our results reproduce this problem in a clinically actionable form: among all VUS, 15.6% were REVEL–AlphaMissense discordant; within missense VUS, this rose to ~22%. Importantly, discordance was not symmetric. It was dominated by REVEL calling pathogenic while AlphaMissense called benign, a structured pattern that would be mishandled by naive averaging.

That directionality is plausible given predictor design choices. REVEL is a curated ensemble meta-predictor trained on pathogenic and rare neutral missense variants, constructed to mitigate overlap with constituent-tool training data [11]. AlphaMissense leverages structure- and sequence-derived representations and is fine-tuned using population frequency-based weak labels rather than clinical assertion datasets [12]. These distinct training paradigms imply different failure modes and biases; our discordance tier operationalizes that insight by treating disagreement as information and routing such variants to orthogonal evidence rather than forcing a direction.

A second, practical uncertainty is incomplete predictor availability. In our VUS set, 48.2% lacked REVEL and/or AlphaMissense, and even within missense the coverage for each was ~87%. Instead of imputing away missingness, we used limited-evidence tiers to prevent overconfident calls based on incomplete annotation. This design choice aligns with the role of ClinVar as an aggregator of heterogeneous submissions and evidentiary standards, valuable but imperfect as a training substrate [9].

Clinical interoperability was a core design goal. We therefore mapped calibrated outputs to the ClinGen PP3/BP4 strength scale proposed by Pejaver et al. (2022), which calibrates continuous predictor scores to evidence strengths using empirical local positive predictive values [10]. Under a combined REVEL × AlphaMissense rule, this yielded 62 PP3_Strong and 84 PP3_Moderate calls, 238 BP4_Supporting calls, and 99 “Conflicting” calls. The close overlap between “Conflicting” PP3/BP4 and our predictor-discordant tier provides an external sanity check that our discordance flag captures what contemporary ACMG calibration frameworks would also treat as non-directional evidence.

This evidence-code output is not merely cosmetic. PP3/BP4 are applied frequently in expert curation and can shift final classifications; analyses of VCEP-curated variants indicate that computational criteria are often decisive near classification boundaries [32]. Because we output evidence codes rather than end-point labels, our framework can be integrated into standard ACMG/AMP rule combining or into quantitative approaches such as Tavtigian’s Bayesian formulation and its naturally scaled point system [27,28]. In practice, this creates a transparent bridge from model probability to evidence weight, while preserving explicit conflict states that should trigger orthogonal evidence gathering.

Although *LDLR* VUS are missense-heavy, non-coding variants remain clinically important because splice disruption is a common pathogenic mechanism. We therefore incorporated splice-region consequence annotation using Ensembl VEP and implemented a conservative splice-rescue rule that reclassified 48 otherwise low-confidence VUS into a low-confidence benign splice tier [18]. This materially reduced the unresolved pool without overstating certainty; it also demonstrates a general principle for clinical ML pipelines: mechanism-specific annotation layers can improve coverage when general pathogenicity predictors are not applicable.

However, categorical “splice region” consequence terms are blunt instruments and may miss deep intronic and cryptic splice effects. A clear next step is to incorporate quantitative splice predictors such as SpliceAI, which predicts splice-altering effects directly from sequence context and has been validated across diverse splice-disrupting variants [22]. Calibrating SpliceAI to PP3/BP4-style evidence would extend our uncertainty-aware framework to a larger fraction of non-coding *LDLR* VUS.

*Internal validation:* The split-half analyses collectively demonstrate that model performance is essentially identical when trained on any random half of the data (Approaches A and B: AUC 0.991–0.994 versus 0.994 on the full set), confirming that the reported nested-CV metrics are not inflated by a fortunate training partition. More importantly, the nested 5× stability analysis (Approach C) revealed that 57.4% of VUS predictions are highly stable (SD < 0.10 across 10 independently trained models), while 13.2% are genuinely unstable (SD ≥ 0.20). The 144 unstable VUS define a model-instability dimension that is orthogonal to the 170 predictor-discordant VUS identified via REVEL × AlphaMissense concordance: a variant may be predictor-concordant yet model-unstable, or vice versa. Both layers represent variants for which functional or segregation evidence would yield the highest diagnostic information gain. We therefore recommend that clinical users of the framework prioritize variants flagged by either uncertainty layer for orthogonal investigation, irrespective of their directional call in the 18-tier framework.

*External validation, clinical positioning, and limitations:* External validation against ClinGen FH-VCEP adjudications strengthens confidence in generalizability because the FH-VCEP applies *LDLR*-specific ACMG/AMP specifications and independently re-adjudicates evidence [7]. When trained on non-expert ClinVar P/LP and B/LB variants and tested on FH-VCEP-reviewed variants, our model achieved ROC-AUC 0.986 overall (0.975 within missense), with high precision at the default threshold. This supports positioning the framework as a triage layer: it accelerates review for concordant high-confidence variants while deliberately isolating discordant and low-evidence cases for orthogonal follow-up.

Our approach is complementary to *LDLR*-specific curated resources such as the LOVD-powered UCL *LDLR* variant database, which curates published variants and highlights records needing additional family or functional evidence, and to the LOVD platform more broadly [33,34]. Operationally, our framework makes a concrete recommendation: discordant missense VUS should be prioritized for segregation, LDL uptake assays, or high-throughput functional assays. Multiplexed assays of variant effect offer a scalable way to resolve missense VUS and to generate high-quality training data for future models [35].

*Epidemiological support from population and sequencing studies:* The clinical plausibility of the proposed *LDLR* VUS prioritization framework is supported by epidemiological and population-scale sequencing evidence linking deleterious *LDLR* variation to quantitative lipid traits and cardiovascular outcomes. Individuals carrying pathogenic or likely pathogenic variants in FH-associated genes, particularly *LDLR*, have consistently been shown to have substantially elevated LDL-C levels and increased risk of premature coronary heart disease (CHD) and coronary artery disease (CAD) compared with non-carriers [6,36,37,38]. In population and health-system cohorts, sequencing-based identification of FH-associated variants has also demonstrated clinical utility by identifying individuals at increased cardiovascular risk who may benefit from earlier lipid-lowering intervention and cascade screening [38]. Large exome and genome-wide sequencing studies, including UK Biobank-based analyses, further confirm that rare loss-of-function and predicted-deleterious missense variants in *LDLR* are among the strongest genetic contributors to LDL-C variation and coronary events [36,37]. Therefore, although our framework does not directly test epidemiological association, the directionality of the pathogenic-leaning and benign-leaning tiers is consistent with established population-level evidence for *LDLR*-mediated FH and cardiovascular risk.

*Translational trajectory:* Looking beyond variant-level classification, the prioritized VUS identified here, particularly predictor-discordant or model-unstable variants, represent candidates for integration with downstream biological and clinical readouts in future studies. Correlation of predicted pathogenicity tiers with quantitative lipid phenotypes (LDL-C, Lp(a), remnant cholesterol), LDLR protein expression or receptor uptake activity, and emerging markers of oxidative stress and endothelial dysfunction associated with *LDLR* haploinsufficiency could provide orthogonal validation of the computational predictions.

*Clinical implementation and resource optimization:* The framework is designed to operate at the point where an *LDLR* VUS is identified through diagnostic sequencing, after variant calling and initial annotation, but prior to or alongside formal expert curation. In familial hypercholesterolemia diagnostics, this corresponds to the interval between return of a molecular result indicating a VUS and the resource-intensive process of expert panel adjudication. By providing calibrated directional evidence and explicit uncertainty flags, the framework enables diagnostic laboratories and variant curation expert panels to prioritize their limited review capacity: high-confidence pathogenic-leaning variants can be fast-tracked for cascade family screening and lipid management intensification, while high-confidence benign-leaning variants can be deprioritized from further workup. Critically, predictor-discordant and model-unstable variants, which the framework explicitly identifies rather than forcing into a binary classification, represent the subset most likely to benefit from additional experimental investment such as functional splicing assays, receptor uptake studies, or extended family segregation analyses. This uncertainty-preserving triage approach optimizes allocation of both clinical and experimental resources by concentrating costly follow-up on the variants where additional data are most likely to resolve classification ambiguity (Figure 10).

*Limitations:* First, ClinVar-derived labels encode heterogeneous evidence standards and potential submission biases [9]. Second, the missense-only model is a structurally limited classifier: after gnomAD-based pseudo-benign expansion the training set still contained only 54 confirmed benign missense examples versus approximately 732 P/LP missense variants. This residual imbalance, together with the small absolute number of benign training instances, contributes to elevated fold-to-fold variance (missense ROC-AUC SD up to 0.022 across outer folds; Table 3) and imposes a hard ceiling on the precision of probability calibration within the missense subset. As a consequence, the missense-only performance metrics reported here should be interpreted as discrimination among clinically curated *LDLR* missense variants and should not be extrapolated, without further validation, to genes with substantially different benign/pathogenic missense ratios, to ultra-rare populations under-represented in gnomAD, or to settings where the prior probability of pathogenicity differs materially from the LDLR/FH context. Expansion of the benign missense training pool through high-throughput functional assays [35] and FH-specific population sequencing remains the most important step toward strengthening the missense-only tier. Third, correlated errors and evaluation circularity remain endemic risks across predictor ecosystems [31]. Finally, *LDLR* copy-number variants were out of scope despite contributing materially to FH molecular diagnoses and requiring orthogonal detection (e.g., MLPA or NGS-based CNV calling) [39]. Prospective validation in diagnostic workflows, expanded and better-curated benign missense sets, incorporation of SpliceAI-calibrated evidence, and systematic functional follow-up of the discordant tier are the most direct routes to strengthening clinical utility.

## 5. Conclusions

We present an interpretable, uncertainty-aware machine learning framework for prioritizing variants of uncertain significance in the *LDLR* gene. The model assigns directional classification to 69% of VUS while explicitly preserving uncertainty, with 15.6% identified as predictor-discordant rather than overclassified. Robust performance in the missense subset (ROC-AUC ~0.97) confirms that predictions are driven by biologically meaningful signals from in silico predictors and amino acid features, rather than trivial consequence effects. Integration of splice-aware annotation further improves classification of non-coding variants, and strong external validation against ClinGen FH Variant Curation Expert Panel data (ROC-AUC ~0.99) supports external validity within the *LDLR*/FH setting. Importantly, mapping outputs to ACMG/AMP-compatible evidence codes enables direct clinical interpretation. Overall, this framework may help prioritize *LDLR* VUS for expert review and downstream validation while preserving uncertainty where evidence is insufficient. It also provides a scalable and clinically aligned approach to reduce VUS uncertainty, prioritize variants for further evaluation, and support precision diagnostics in familial hypercholesterolemia.

## Figures and Tables

**Table 1:** VUS prioritization framework full tier definitions. Rules are evaluated from top to bottom: splice rescue takes priority over predictor discordance, which takes priority over the standard probability tiers. p = calibrated P(pathogenic) from the all-variant XGBoost model after probability calibration using isotonic regression on the full training set.

Tiers	Tier	Probability Range	Additional Condition
1	P_LP_splice_evidence	p ≥ 0.5	non-coding + splice-involved
2	B_LB_splice_low	p < 0.5	non-coding + splice-involved
3	Predictor_Discordant_VUS	any	REVEL & AM disagree
4	P_LP_high_concordant	p ≥ 0.90	concordant_pathogenic
5	P_LP_high	p ≥ 0.90	—
6	P_LP_moderate	0.80 ≤ p < 0.90	predictors available
7	P_LP_moderate_limited_evidence	0.80 ≤ p < 0.90	no REVEL or AM
8	P_LP_predictor_supported	0.65 ≤ p < 0.80	concordant_pathogenic
9	P_LP_low	0.65 ≤ p < 0.80	predictors available
10	P_LP_low_limited_evidence	0.65 ≤ p < 0.80	no REVEL or AM
11	B_LB_high_concordant	p ≤ 0.10	concordant_benign
12	B_LB_high	p ≤ 0.10	—
13	B_LB_moderate	0.10 < p ≤ 0.20	predictors available
14	B_LB_moderate_limited_evidence	0.10 < p ≤ 0.20	no REVEL or AM
15	B_LB_predictor_supported	0.20 < p ≤ 0.35	concordant_benign
16	B_LB_low	0.20 < p ≤ 0.35	predictors available
17	B_LB_low_limited_evidence	0.20 < p ≤ 0.35	no REVEL or AM
18	Unresolved_VUS	0.35 < p < 0.65	no rescuing evidence

**Table 2:** Cohort composition of *LDLR* variants from ClinVar (*LDLR*_3962) after CLNSIG normalization. Pathogenic/likely pathogenic (P_LP) and benign/likely benign (B_LB) variants (n = 2870) were used for model training, while variants of uncertain significance (VUS; n = 1092) were reserved for prediction.

ClinVar Significance	n	%
Pathogenic/Likely_pathogenic (P_LP)	1880	47.5
Benign/Likely_benign (B_LB)	990	25.0
Variant of uncertain significance (VUS)	1092	27.6
Total	3962	100.0

For external validation (Section 3.12), the model was retrained on 2651 non-expert-panel P_LP/B_LB variants (1698 P_LP + 953 B_LB), with 219 expert panel-reviewed variants (182 P_LP, 37 B_LB) held out as an independent test set, and 275 expert-panel VUS used to evaluate classification behavior.

**Table 3:** Nested 5 × 3 cross-validation performance for three model classes. Mean ± SD across 5 outer folds, with hyperparameters selected by 3-fold RandomizedSearchCV on the inner loop (20 sampled configurations for RF/XGBoost, 10 for SVM). SVM uses a Pipeline (StandardScaler, SVC) with RBF/linear kernels and class_weight = “balanced”. PR-AUC, F1 and Brier are reported as fold means. See Figure 2 and Figure 3 for visualization.

Model	Stage	Mean ROC-AUC	Mean PR-AUC	Mean F1	Mean Brier
RandomForest	All-variant	0.9926 ± 0.0024	0.9960	0.9792	0.0227
XGBoost	All-variant	0.9943 ± 0.0018	0.9971	0.9793	0.0220
RandomForest	Missense-only	0.9790 ± 0.0134	0.9984	0.9694	0.0340
XGBoost	Missense-only	0.9732 ± 0.0223	0.9980	0.9639	0.0486
SVM	All-variant	0.9894 ± 0.0054	0.9943	0.9747	0.0266
SVM	Missense-only	0.9713 ± 0.0158	0.9979	0.9749	0.0347

**Table 4:** Final VUS classification by direction. Final classification of VUS by direction based on calibrated probabilities. Pathogenic and benign directions aggregate all confidence tiers, while discordant variants reflect disagreement between REVEL and AlphaMissense, and unresolved variants correspond to the abstention probability range (0.35 < p < 0.65).

Direction	n	% of VUS
Pathogenic-leaning	485	44.4
Benign-leaning	268	24.5
Discordant	170	15.6
Unresolved	169	15.5
Total	1092	100.0

**Table 5:** REVEL × AlphaMissense predictor concordance. Predictor concordance categories for all 1092 VUS based on REVEL and AlphaMissense thresholds. Concordant categories require agreement in direction, while discordant categories capture opposing predictions. Variants lacking one or both predictors are classified as insufficient_predictors.

Predictor Concordance Category	n	% of VUS
concordant_pathogenic	131	12.0
concordant_benign	61	5.6
discordant_REVEL_path_AM_benign	168	15.4
discordant_AM_path_REVEL_benign	2	0.2
both_uncertain	204	18.7
insufficient_predictors	526	48.2
Total	1092	100.0

**Table 6:** Feature-group ablation analysis showing the impact of removing individual feature groups on model performance. ΔAUC = AUC_full − AUC_dropped; positive values indicate a reduction in performance upon feature removal. Models were evaluated using fixed-hyperparameter XGBoost with 5-fold StratifiedKFold. The in silico predictor group is the dominant contributor to missense-level performance, while the consequence group primarily drives all-variant performance.

Feature Group Dropped	# Number of Features Dropped	AUC All-Variant	ΔAUC All	AUC Missense	ΔAUC Missense
(none: full)	0	0.9939	+0.0000	0.9589	+0.0000
in_silico	16	0.9900	+0.0039	0.8046	+0.1543
allele_frequency	14	0.9933	+0.0006	0.9597	−0.0008
aa_biochemistry	7	0.9937	+0.0002	0.9612	−0.0023
splice	1	0.9939	−0.0000	0.9585	+0.0004
consequence	21	0.9724	+0.0215	0.9591	−0.0002
interactions	4	0.9940	−0.0001	0.9542	+0.0047
sequence	6	0.9941	−0.0002	0.9603	−0.0013

**Table 7:** Distribution of combined PP3/BP4 computational evidence codes for 1092 VUS using calibrated REVEL and AlphaMissense thresholds. The combined code reflects the stronger of concordant signals, while “Conflicting” denotes disagreement between predictors and corresponds to the Predictor_Discordant_VUS category. “NA” indicates missing predictor data.

Combined PP3/BP4 Evidence Code	n	% of VUS
NA	514	47.1
BP4_Supporting	238	21.8
Conflicting	99	9.1
PP3_Moderate	84	7.7
PP3_Strong	62	5.7
PP3_Supporting	60	5.5
Indeterminate	20	1.8
BP4_Moderate	15	1.4

**Table 8:** External validation against ClinGen FH-VCEP. Performance of the all-variant XGBoost model evaluated on 219 ClinGen FH-VCEP expert panel-reviewed *LDLR* variants. The model was trained exclusively on 2651 non-expert-panel P_LP/B_LB variants. Metrics are reported at the default probability threshold (0.5).

Subset	n	P_LP	B_LB	ROC-AUC	PR-AUC	F1	Brier	Prec.	Sens.
all (FH-VCEP held-out)	219	182	37	0.9861	0.9971	0.9602	0.0568	0.994	0.929
missense (FH-VCEP held-out)	161	151	10	0.9748	0.9982	0.9695	0.0456	0.993	0.947
non-missense (FH-VCEP held-out)	58	31	27	0.9898	0.9904	0.9123	0.0877	1.000	0.839

**Table 9:** Comparative performance on FH-VCEP held-out validation. Performance of REVEL alone, AlphaMissense alone, their combination (logistic regression and XGBoost), and the full 45-feature calibrated pipeline. In Panel B, single-predictor and two-predictor models were evaluated on the 153 missense variants with both REVEL and AlphaMissense scores available, whereas the full pipeline was evaluated on all 161 FH-VCEP missense variants by leveraging median imputation for missing predictor values. Bold indicates best-performing model per metric.

**Panel A: All Variants (n = 219)**						
**Model**	**ROC-AUC**	**PR-AUC**	**F1**	**Brier**	**Precision**	**Recall**
REVEL-only (LR)	0.567	0.875	0.905	0.180	0.850	0.967
AlphaMissense-only (LR)	0.543	0.862	0.908	0.187	0.831	1.000
REVEL + AlphaMissense (LR)	0.497	0.844	0.907	0.185	0.854	0.967
REVEL + AlphaMissense (XGBoost)	0.850	0.959	0.908	0.106	0.851	0.973
**Full pipeline (45 features)**	**0.986**	**0.997**	**0.960**	**0.057**	**0.994**	**0.929**
**Panel B: Missense-Only Subset (n = 153)**						
**Model**	**ROC-AUC**	**PR-AUC**	**F1**	**Brier**	**Precision**	**Recall**
REVEL-only (LR)	0.947	0.996	0.972	0.146	0.979	0.965
AlphaMissense-only (LR)	0.978	0.999	0.970	0.159	0.941	1.000
REVEL + AlphaMissense (LR)	0.970	0.998	0.975	0.154	0.986	0.965
REVEL + AlphaMissense (XGBoost)	0.933	0.996	0.969	0.055	0.972	0.965
**Full pipeline (45 features)**	**0.975**	**0.998**	**0.969**	**0.046**	**0.993**	**0.947**

## Data Availability

All code and analysis pipelines used in this study are publicly available at https://github.com/NaderHMC-lab/LDLR-VUS-classifier (accessed on 16 April 2026). The repository includes scripts for data processing, model training, evaluation, and figure generation. Input data were derived from the ClinVar dataset (clinvar_all_classes_genes_cmg.xlsx, sheet *LDLR*_3962) and all outputs are generated within the repository. Analyses are fully reproducible using the provided scripts with fixed random seeds (SEED = 42). API-based annotations (myvariant.info and Ensembl VEP REST) are cached during the initial run, enabling subsequent executions without network dependency.

## Supplementary Materials

The following supporting information can be downloaded at https://www.mdpi.com/article/10.3390/genes17091057/s1, Figure S1: Approach A—Split-half performance. (a) ROC-AUC and F1 for each labelled half compared with the full-data nested-CV baseline. (b) VUS classification distribution from the two split-half models, showing a pattern consistent with the full-data pipeline; Figure S2: Approach B—Cross-prediction performance. (a) ROC-AUC for each cross-prediction direction, with all-variant (dark blue) and missense-only (light blue) shown separately. (b) Confusion matrix for all 2870 labelled variants under bidirectional cross-prediction (TP = 1,825; TN = 964; FP = 26; FN = 55); Figure S3: Approach C—Nested 5× split-half stability analysis. (a) Histogram of per-VUS standard deviation across 10 model probabilities; dashed lines mark stability tier boundaries. (b) Stability tier distribution (SD-based). (c) Consensus tier distribution from 10-model majority voting. (d) Per-model held-out ROC-AUC for all 10 models; dashed line = mean AUC (0.992); Table S1: Feature engineering schema (45 raw to 68 encoded features); Table S2: Variant-level VUS predictions with ACMG/AMP evidence mapping; Table S3: ACMG/AMP evidence tier cross-tabulation summary; Table S4: Approach A—Nested 5-fold cross-validation performance on each labelled half. Each half contains 940 P_LP and 495 B_LB variants (1435 total). Results are comparable to the full-data nested CV (ROC-AUC 0.9943 for XGBoost, Table 2); Table S5: Approach B—Out-of-sample cross-prediction between the two labelled halves. Each labelled variant is predicted exclusively by a model trained on the other half. Missense-only rows provide the clinically critical within-consequence performance estimate; Table S6: Approach C—Stability-tier distribution for the 1,092 VUS, derived from ten independently trained calibrated XGBoost models (5 splits × 2 halves). SD is the standard deviation of the 10 per-VUS probabilities. Unstable variants (SD ≥ 0.20) define a continuous model-instability dimension that is orthogonal to predictor concordance (Section 2.7); Table S7: Approach C—Consensus tier distribution from the 10-model voting rule. P_LP_unanimous = all 10 models called P(pathogenic) ≥ 0.5; B_LB_unanimous = all 10 models called P(pathogenic) < 0.5; Unstable_flag = SD ≥ 0.20 regardless of direction; Table S8: External validation predictions on the held-out ClinGen FH-VCEP variants; Table S9: Summary comparison of the three internal validation strategies applied to the LDLR XGBoost classifier. Strategy A trains and evaluates within each half independently (nested 5-fold cross-validation); Strategy B cross-predicts between halves, producing fully out-of-sample predictions for all 2870 labelled variants; Strategy C trains 10 calibrated models across five 50/50 stratified splits (seeds 42–46) and aggregates predictions per VUS to quantify prediction stability and consensus. Metrics are reported at the default probability threshold of 0.5. AUC = area under the receiver-operating-characteristic curve; F1 = harmonic mean of precision and recall; Brier = mean squared error of calibrated probabilities; SD = standard deviation across the 10 models; n.a. = not applicable for this strategy.

## Abbreviations

The following abbreviations are used in this manuscript:

FH	Familial Hypercholesterolemia
LDLR	Low-Density Lipoprotein Receptor
VUS	Variants of Uncertain Significance
ACMG	American College of Medical Genetics and Genomics
AMP	Association for Molecular Pathology
PP3	Pathogenic Supporting Evidence (computational)
BP4	Benign Supporting Evidence (computational)
FH-VCEP	Familial Hypercholesterolemia Variant Curation Expert Panel
VEP	Variant Effect Predictor
SNV	Single-Nucleotide Variant
MC	Molecular Consequence
CLNSIG	Clinical Significance
CLNREVSTAT	Clinical Review Status
P_LP	Pathogenic/Likely Pathogenic
B_LB	Benign/Likely Benign
AF	Allele Frequency
ESP	Exome Sequencing Project
ExAC	Exome Aggregation Consortium
gnomAD	Genome Aggregation Database
REVEL	Rare Exome Variant Ensemble Learner
CADD	Combined Annotation Dependent Depletion
SIFT	Sorting Intolerant from Tolerant
SHAP	Shapley Additive Explanations
ML	Machine Learning
RF	Random Forest
SVM	Support Vector Machine
ROC-AUC	Receiver Operating Characteristic Area Under the Curve
PR-AUC	Precision–Recall Area Under the Curve
F1	F1 Score
