# Supplementary material for: Machine Learning Triage of LDLR Variants of Uncertain Significance Using Predictor Concordance and ACMG-Aligned Evidence Mapping
Source: Genes (Basel). 2026 Aug 31;17(9):1057. doi: 10.3390/genes17091057 (PMC13606661; doi:10.3390/genes17091057)
Supplement: Supplementary file 1 [file genes-17-01057-s001.zip › Supplementary_Figures.pdf]

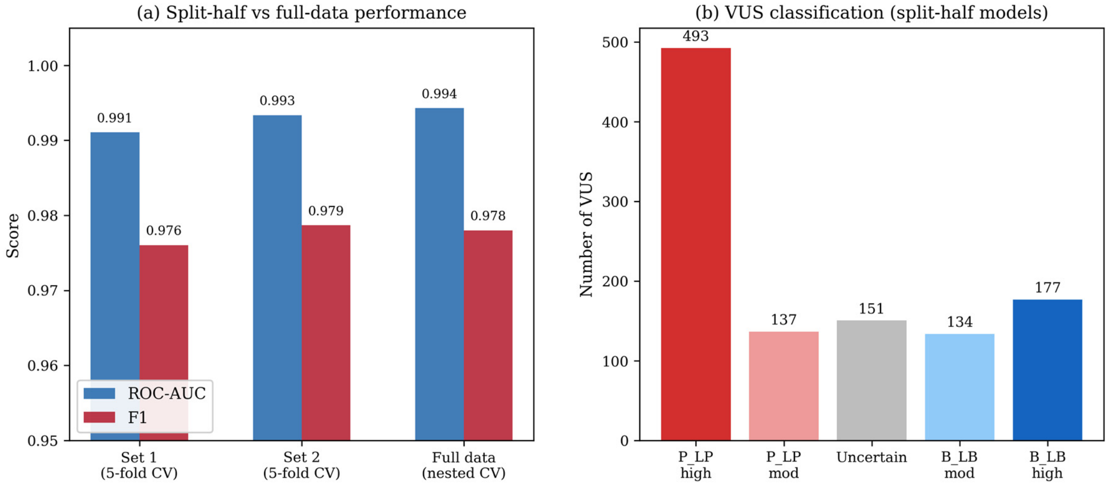

Figure S1. Approach A — Split-half performance. (a) ROC-AUC and F1 for each labelled half compared with the full-data nested-CV baseline. (b) VUS classification distribution from the two split-half models, showing a pattern consistent with the full-data pipeline.

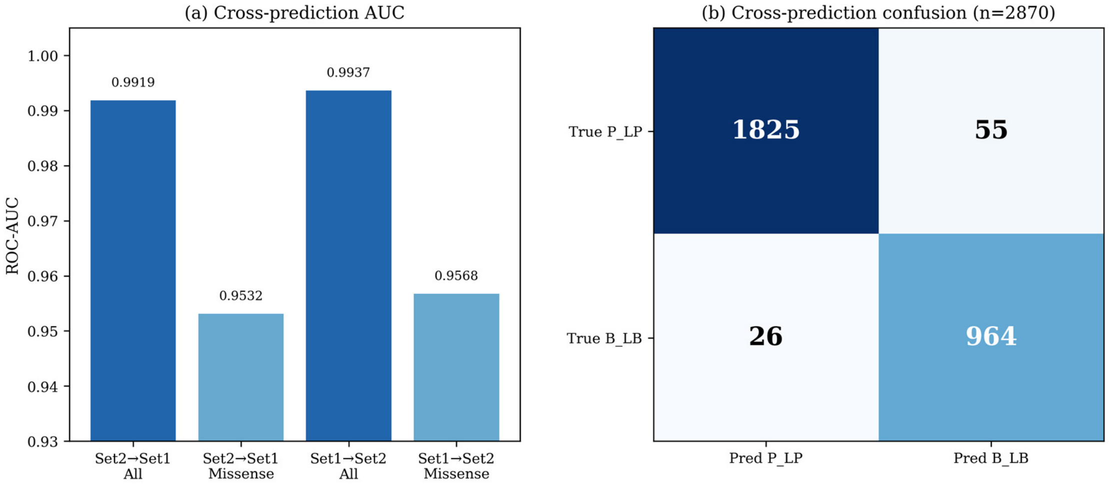

Figure S2. Approach B — Cross-prediction performance. (a) ROC-AUC for each cross-prediction direction, with all-variant (dark blue) and missense-only (light blue) shown separately. (b) Confusion matrix for all 2,870 labelled variants under bidirectional cross-prediction (TP = 1,825; TN = 964; FP = 26; FN = 55).

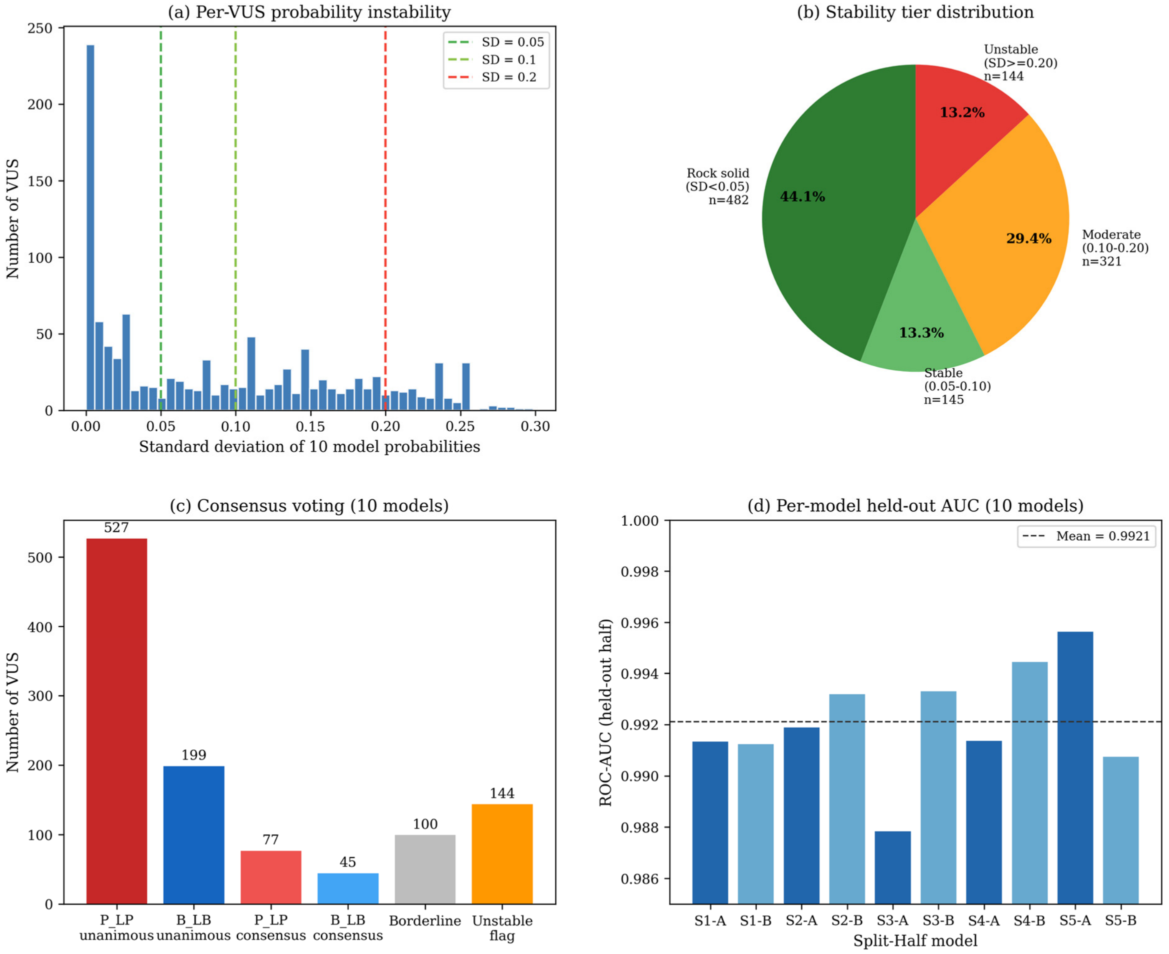

Figure S3. Approach C — Nested 5× split-half stability analysis. (a) Histogram of per-VUS standard deviation across 10 model probabilities; dashed lines mark stability tier boundaries. (b) Stability tier distribution (SD-based). (c) Consensus tier distribution from 10-model majority voting. (d) Per-model held-out ROC-AUC for all 10 models; dashed line = mean AUC (0.992).
