# Supplementary material for: Machine Learning Triage of LDLR Variants of Uncertain Significance Using Predictor Concordance and ACMG-Aligned Evidence Mapping
Source: Genes (Basel). 2026 Aug 31;17(9):1057. doi: 10.3390/genes17091057 (PMC13606661; doi:10.3390/genes17091057)
Supplement: Supplementary file 1 [file genes-17-01057-s001.zip › Table_S1.pdf]

Table S1. Feature Engineering: 45 Raw → 68 Encoded Features. The pipeline constructs 45 base features from the annotated variant data. Three of these are categorical (text-valued) and are expanded into binary indicator columns via one-hot encoding, producing 68 final features used by the ML models.

Summary of 45 → 68 Expansion

Feature Group	Pre-encode Columns	Categorical?	Post-encode Columns	Net Change
Sequence	6	No	6	—
Allele Frequency (numeric)	8	No	8	—
Allele Frequency Bin	1	Yes → 5 bins	5	+4
Consequence	1	Yes → 14 types	14	+13
Variant Class	1	Yes → 7 types	7	+6
In Silico Predictors	14	No	14	—
Amino-acid Biochemistry	7	No	7	—
Splice	1	No	1	—
Allele Origin	1	No	1	—
Interaction	4	No	4	—
**TOTAL**	**45**	**3 categorical**	**68**	**+23**

Complete 68-Feature Listing

#	Feature Name	Group	Type	Description
1	ref_len	Sequence	Numeric	Length of REF allele
2	alt_len	Sequence	Numeric	Length of ALT allele
3	len_diff	Sequence	Numeric	ALT length − REF length
4	is_snv	Sequence	Binary	1 if single-nucleotide variant
5	is_indel	Sequence	Binary	1 if insertion/deletion
6	transition	Sequence	Binary	1 if transition (A↔G, C↔T)
7	af_esp	Allele Freq	Numeric	AF from ESP database
8	af_esp_measured	Allele Freq	Binary	1 if ESP had data for this

				variant
9	af_exac	Allele Freq	Numeric	AF from ExAC database
10	af_exac_measured	Allele Freq	Binary	1 if ExAC had data
11	af_tgp	Allele Freq	Numeric	AF from 1000 Genomes
12	af_tgp_measured	Allele Freq	Binary	1 if 1000G had data
13	af_max	Allele Freq	Numeric	Max AF across ESP/ExAC/1000G
14	af_max_log	Allele Freq	Numeric	log10(af_max + 1e-8)
15	any_af_measured	Allele Freq	Binary	1 if any AF source had data
16	af_bin_not_measured	AF Bin (OH)	Binary	No AF database had data
17	af_bin_ultra_rare	AF Bin (OH)	Binary	AF < 0.0001
18	af_bin_rare	AF Bin (OH)	Binary	0.0001 ≤ AF < 0.001
19	af_bin_low_freq	AF Bin (OH)	Binary	0.001 ≤ AF < 0.01
20	af_bin_common	AF Bin (OH)	Binary	AF ≥ 0.01
21	consequence_missense	Consequence (OH)	Binary	Missense variant
22	consequence_nonsense	Consequence (OH)	Binary	Stop gained
23	consequence_frameshift	Consequence (OH)	Binary	Frameshift variant
24	consequence_synonymous	Consequence (OH)	Binary	Synonymous variant
25	consequence_intronic	Consequence (OH)	Binary	Intronic variant
26	consequence_splice_donor	Consequence (OH)	Binary	Splice donor site
27	consequence_splice_acceptor	Consequence (OH)	Binary	Splice acceptor site
28	consequence_non_coding	Consequence (OH)	Binary	Non-coding variant
29	consequence_3_prime_UTR	Consequence (OH)	Binary	3' UTR variant
30	consequence_5_prime_UTR	Consequence (OH)	Binary	5' UTR variant
31	consequence_inframe_deletion	Consequence (OH)	Binary	In-frame deletion
32	consequence_inframe_insertion	Consequence (OH)	Binary	In-frame insertion
33	consequence_other	Consequence (OH)	Binary	Other consequence
34	consequence_unknown	Consequence (OH)	Binary	Unknown consequence
35	variant_class_single_nucleotide_variant	Variant Class (OH)	Binary	ClinVar: SNV
36	variant_class_deletion	Variant Class (OH)	Binary	ClinVar: deletion
37	variant_class_insertion	Variant Class (OH)	Binary	ClinVar: insertion
38	variant_class_indel	Variant Class (OH)	Binary	ClinVar: indel
39	variant_class_duplication	Variant Class (OH)	Binary	ClinVar: duplication

40	variant_class_inversion	Variant Class (OH)	Binary	ClinVar: inversion
41	variant_class_microsatellite	Variant Class (OH)	Binary	ClinVar: microsatellite
42	revel_score	In Silico	Numeric	REVEL pathogenicity score (0–1)
43	cadd_phred	In Silico	Numeric	CADD Phred-scaled score
44	polyphen2_hdiv	In Silico	Numeric	PolyPhen-2 HumDiv score (0–1)
45	sift_score	In Silico	Numeric	SIFT score (0–1, lower = damaging)
46	sift_inverted	In Silico	Numeric	1 − SIFT (higher = damaging)
47	alphamissense_score	In Silico	Numeric	AlphaMissense score (0–1)
48	mutationtaster_rankscore	In Silico	Numeric	MutationTaster rank score
49	consensus_pathogenicity	In Silico	Numeric	Mean of normalized predictors
50	n_predictors_available	In Silico	Count	How many of 5 predictors had data
51	revel_score_available	In Silico	Binary	1 if REVEL score exists
52	cadd_phred_available	In Silico	Binary	1 if CADD score exists
53	polyphen2_hdiv_available	In Silico	Binary	1 if PolyPhen-2 score exists
54	sift_score_available	In Silico	Binary	1 if SIFT score exists
55	alphamissense_score_available	In Silico	Binary	1 if AlphaMissense score exists
56	grantham_dist	AA Biochem	Numeric	Grantham distance (0–215)
57	hydro_change	AA Biochem	Numeric	Δ hydrophobicity (Kyte-Doolittle)
58	abs_hydro_change	AA Biochem	Numeric	|Δ hydrophobicity|
59	charge_change	AA Biochem	Numeric	Δ amino-acid charge
60	has_aa_change	AA Biochem	Binary	1 if amino acid actually changes
61	loses_cysteine	AA Biochem	Binary	1 if Cys → non-Cys (breaks disulfide)
62	to_proline	AA Biochem	Binary	1 if non-Pro → Pro (breaks helix)
63	is_splice_variant_vep	Splice	Binary	1 if VEP flagged splice involvement
64	origin	Origin	Code	ClinVar ORIGIN code (germline/somatic)
65	rare_missense	Interaction	Binary	Rare + missense

66	rare_lof	Interaction	Binary	Rare + loss-of-function
67	revel_high_rare	Interaction	Binary	REVEL ≥ 0.7 + rare/not-measured
68	revel_low_common	Interaction	Binary	REVEL ≤ 0.3 + common/low-freq

OH = one-hot encoded (expanded from a single categorical column). The three categorical features (af_bin, consequence, variant_class) expand from 3 columns to 26 columns (5 + 14 + 7), accounting for the 45 → 68 increase.
