# Supplementary material for: Machine Learning Triage of LDLR Variants of Uncertain Significance Using Predictor Concordance and ACMG-Aligned Evidence Mapping
Source: Genes (Basel). 2026 Aug 31;17(9):1057. doi: 10.3390/genes17091057 (PMC13606661; doi:10.3390/genes17091057)
Supplement: Supplementary file 1 [file genes-17-01057-s001.zip › Table_S4.pdf]

Table S4. Approach A — Nested 5-fold cross-validation performance on each labelled half. Each half contains 940 P_LP and 495 B_LB variants (1,435 total). Results are comparable to the full-data nested CV (ROC-AUC 0.9943 for XGBoost, Table 2).

ss

Set	n	P_LP	B_LB	ROC-AUC	PR-AUC	F1	Brier
Set 1 (5-fold CV)	1,435	940	495	0.9911	0.9949	0.9760	0.0250
Set 2 (5-fold CV)	1,435	940	495	0.9934	0.9968	0.9787	0.0233
Full data (ref.)	2,870	1,880	990	0.9943	—	0.978	—
