# Supplementary material for: Machine Learning Triage of LDLR Variants of Uncertain Significance Using Predictor Concordance and ACMG-Aligned Evidence Mapping
Source: Genes (Basel). 2026 Aug 31;17(9):1057. doi: 10.3390/genes17091057 (PMC13606661; doi:10.3390/genes17091057)
Supplement: Supplementary file 1 [file genes-17-01057-s001.zip › Table_S5.pdf]

Table S5. Approach B — Out-of-sample cross-prediction between the two labelled halves. Each labelled variant is predicted exclusively by a model trained on the other half. Missense-only rows provide the clinically critical within-consequence performance estimate.

ss

Training → Test	Subset	n	ROC-AUC	F1	Brier	Prec.	Sens.
Set 2 → Set 1	All variants	1,435	0.9919	0.9748	0.0256	0.984	0.966
Set 2 → Set 1	Missense only	373	0.9532	0.9788	0.0290	0.977	0.980
Set 1 → Set 2	All variants	1,435	0.9937	0.9818	0.0197	0.988	0.976
Set 1 → Set 2	Missense only	403	0.9568	0.9802	0.0306	0.981	0.979
