# Supplementary material for: Machine Learning Triage of LDLR Variants of Uncertain Significance Using Predictor Concordance and ACMG-Aligned Evidence Mapping
Source: Genes (Basel). 2026 Aug 31;17(9):1057. doi: 10.3390/genes17091057 (PMC13606661; doi:10.3390/genes17091057)
Supplement: Supplementary file 1 [file genes-17-01057-s001.zip › Table_S6.pdf]

Table S6. Approach C — Stability-tier distribution for the 1,092 VUS, derived from ten independently trained calibrated XGBoost models (5 splits × 2 halves). SD is the standard deviation of the 10 per-VUS probabilities. Unstable variants (SD ≥ 0.20) define a continuous model-instability dimension that is orthogonal to predictor concordance (Section 2.7).

Stability tier	SD range	n	% of VUS
Highly-stable	< 0.05	482	44.1%
Stable	0.05 – 0.10	145	13.3%
Moderate	0.10 – 0.20	321	29.4%
Unstable	≥ 0.20	144	13.2%
**Total**		**1,092**	**100.0%**
