# Supplementary material for: Machine Learning Triage of LDLR Variants of Uncertain Significance Using Predictor Concordance and ACMG-Aligned Evidence Mapping
Source: Genes (Basel). 2026 Aug 31;17(9):1057. doi: 10.3390/genes17091057 (PMC13606661; doi:10.3390/genes17091057)
Supplement: Supplementary file 1 [file genes-17-01057-s001.zip › Table_S7.pdf]

Table S7. Approach C — Consensus tier distribution from the 10-model voting rule. P_LP_unanimous = all 10 models called P(pathogenic) ≥ 0.5; B_LB_unanimous = all 10 models called P(pathogenic) < 0.5; Unstable_flag = SD ≥ 0.20 regardless of direction.

Consensus tier	Rule	n	% of VUS
P_LP_unanimous	10/10 models pathogenic	527	48.3%
B_LB_unanimous	0/10 models pathogenic	199	18.2%
P_LP_consensus	8–9/10 pathogenic, mean ≥ 0.65	77	7.1%
B_LB_consensus	1–2/10 pathogenic, mean ≤ 0.35	45	4.1%
Borderline	Mixed, SD < 0.20	100	9.2%
Unstable_flag	SD ≥ 0.20	144	13.2%
**Total**		**1,092**	**100.0%**

s
