# Supplementary material for: Machine Learning Triage of LDLR Variants of Uncertain Significance Using Predictor Concordance and ACMG-Aligned Evidence Mapping
Source: Genes (Basel). 2026 Aug 31;17(9):1057. doi: 10.3390/genes17091057 (PMC13606661; doi:10.3390/genes17091057)
Supplement: Supplementary file 1 [file genes-17-01057-s001.zip › Table_S9.pdf]

Table S9. Summary comparison of the three internal validation strategies applied to the *LDLR* XGBoost classifier. Strategy A trains and evaluates within each half independently (nested 5-fold cross-validation); Strategy B cross-predicts between halves, producing fully out-of-sample predictions for all 2,870 labelled variants; Strategy C trains 10 calibrated models across five 50/50 stratified splits (seeds 42–46) and aggregates predictions per VUS to quantify prediction stability and consensus. Metrics are reported at the default probability threshold of 0.5. AUC = area under the receiver-operating-characteristic curve; F1 = harmonic mean of precision and recall; Brier = mean squared error of calibrated probabilities; SD = standard deviation across the 10 models; n.a. = not applicable for this strategy.

Attribute	Strategy A — Split-half nested CV	Strategy B — Cross-prediction between halves	Strategy C — Five 50/50 stratified splits (10 models)
**Design**	Independent nested 5-fold CV on each labelled half (Set 1, Set 2)	Model trained on one half, applied to the opposite half; pooled over both directions	Five 50/50 stratified splits (seeds 42–46); 10 calibrated XGBoost models trained on the held-in halves
**Training set per model**	~1,435 labelled variants (one half)	~1,435 labelled variants (one half)	~1,435 labelled variants per model (10 models)
**Evaluation set**	Held-out folds within each half	All 2,870 labelled variants, fully out-of-sample	Held-out half for each split (pooled across seeds)
**ROC-AUC (labelled)**	Set 1: 0.9911; Set 2: 0.9934	Set 2 → Set 1: 0.9919; Set 1 → Set 2: 0.9937	Mean 0.9921 (range 0.9879–0.9957)
**F1 (labelled, threshold = 0.5)**	Set 1: 0.976; Set 2: 0.979	Set 2 → Set 1: 0.975; Set 1 → Set 2: 0.982	Consistent with Strategies A and B across all 10 models
**Brier score**	Set 1: 0.025; Set 2: 0.023	n.a. (combined out-of-sample)	n.a. (aggregated across models)
**Missense-restricted AUC**	0.9732 (nested CV)	0.9532 (n = 373); 0.9568 (n = 403)	n.a. (reported in aggregate)
**Pooled labelled accuracy**	n.a.	97.2% (2,789 / 2,870); 1,825 TP, 964 TN, 26 FP, 55 FN	n.a.
**VUS predictions (n = 1,092)**	493 P_LP_high, 137 P_LP_moderate, 177 B_LB_high, 134 B_LB_moderate, 151 uncertain (Table S4, Figure S1)	Not applied to VUS (labelled-only evaluation)	Stability: mean SD 0.089, median 0.074; 627 stable (57.4%), 144 unstable (13.2%) (Table S6); consensus: 527

			unanimous P (48.3%), 199 unanimous B (18.2%), 122 near-unanimous (11.2%); 848 strong-consensus VUS (77.7%) (Table S7)
**Primary purpose**	Confirm that VUS predictions do not depend on the full 2,870-variant training set	Provide fully out-of-sample labelled-variant performance via reciprocal prediction	Quantify per-VUS prediction stability and inter-model consensus across seeds
**Supporting figures/tables**	Table S4, Figure S1	Table S5, Figure S2	Tables S6, S7; Figure S3
**Reference benchmark**	Full-data XGBoost ROC-AUC = 0.9943 (Table 2)	Full-data XGBoost ROC-AUC = 0.9943 (Table 2)	Full-data XGBoost ROC-AUC = 0.9943 (Table 2)
